# Magnetic N-doped CNT stabilized Cu_2_O as a catalyst for *N*-arylation of nitriles and aryl halides in a biocompatible deep eutectic solvent†

**DOI:** 10.1039/d5ra00849b

**Published:** 2025-03-18

**Authors:** Mohadeseh Alizadeh, Arefe Salamatmanesh, Masoumeh Jadidi Nejad, Akbar Heydari

**Affiliations:** a Chemistry Department, Tarbiat Modares University P.O. Box 14155-4838 Tehran Iran heydar_a@modares.ac.ir +98-21-82883455 +98-21-82883444; b Department of Chemistry, Isfahan University of Technology Isfahan 84156-83111 Iran m.jadidinejad@iut.ac.ir +98-31-33913261

## Abstract

This study documented the hydrolysis of nitriles by copper(i) oxide immobilized on nitrogen-doped carbon nanotubes (N-CNT/Fe_3_O_4_–Cu_2_O) to yield corresponding amides in the presence of a deep eutectic solvent (ChOH/Gly). Furthermore, the aforementioned catalyst can facilitate the coupling reaction between aryl halides and amides derived by nitrile hydrolysis. Consequently, the integration of two copper-catalyzed processes can efficiently provide *N*-aryl amides. Choline hydroxide in a deep eutectic solvent serves as a cost-effective organic catalyst in nitrie hydrolysis by forming hydrogen bonds with nitrile, thereby activating it. The catalyst generated higher to satisfactory product yields. One of the special benefits of the catalyst is that it can be restored through the addition of an external magnetic field.

## Abstract 2

This study documented the hydrolysis of nitriles by copper(i) oxide immobilized on nitrogen-doped carbon nanotubes (N-CNT/Fe_3_O_4_–Cu_2_O) to yield corresponding amides in the presence of a deep eutectic solvent (ChOH/Gly).

## Graphical abstract

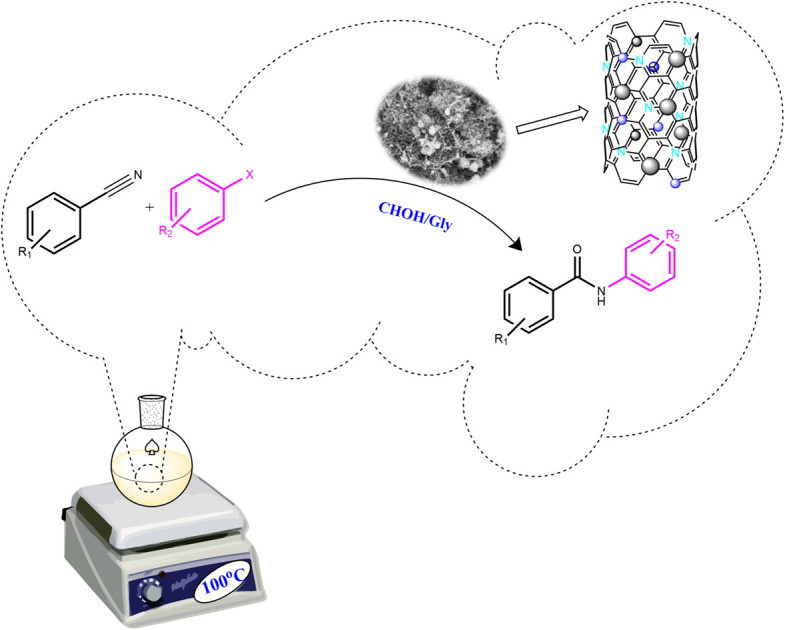

## 1 Introduction

*N*-Arylamides are significant compounds that are frequently used in various fields of materials science and medicinal chemistry. They can also serve as important building blocks for high-performance materials and as possible precursors in the production of organic compounds.^1–6^ The coupling of a carboxylic acid (with coupling reagents like carbodiimide) with an amine is part of the conventional procedure for amide synthesis.^7^ This process has significant environmental issues by nature because it generates a stoichiometric amount of waste products. The use of aldehydes as amide-producing substrates is one of the main challenges in the formation of amide compounds. Depending on the method employed, this transformation may occur immediately or slowly. Aldehydes have been converted into their corresponding amides using three different methods. The most common process is the oxidation of hemiaminals, although there are other paths that are also worth talking about, such as the creation of a C(O)–X intermediate and C(O)–H insertion.^8^ The Beckmann reaction (BKR) is the most often used technique for amide bond production among the various rearrangements that are accessible. According to a classical interpretation, the Beckmann reaction is the rearrangement of an oxime that produces an ion of nitrilium that is trapped by a nucleophile. The transition-metal-catalyzed processes are believed to progress through a dehydration/hydration sequence by producing a unique nitrile intermediate.^9,10^ It appears that several precious transition metal catalysts (such as Rh,^11^ Ru,^12^ Ir,^13^ and Pd^14^) were effectively used for the atom-efficient synthesis of primary amides from aldoximes. The utilization of copper catalysts for these conversions has attracted significant attention due to its economic importance. Jawahar and coworkers effectively transformed aldoximes into primary amides *via* a Cu(OTf)_2_ catalyst.^15^ The BKR reaction has been catalyzed by Cu(OTf)_2_ in two separate ways. Firstly, it facilitates the formation of the transient ketoxime intermediate as it functions as a mild Lewis acid. Subsequently, the R′ group migrates to the nitrogen, where it is ultimately catalyzed by copper, producing the Beckmann product. Copper-catalyzed direct C–N bond formation between benzamides and aryl halides served as the basis for several previous synthetic works using benzanilides.^16^ Large-scale usage of these processes is restricted by the frequent requirement for costly polar solvents like hazardous dioxane, DMSO, or DMF. However, nitriles, which are the precursors to amides, can also be utilized as the substrate in the traditional Ritter reaction to create *N*-substituted amides.^17^ Copper-assisted nitrile hydrolysis into corresponding amides in water has been reported by a few researchers.^18^ As was previously indicated, copper can also accelerate the coupling of amides and aryl halides.^19^ An exceptional investigation was carried out by Xiang and co-workers^20^ about the sequential hydrolysis/coupling reaction between aryl iodides and simple nitriles, which was catalyzed by copper. Xiang's method is not economically viable due to its utilization of significant amounts of nitriles as both solvent and reactant.

Ionic liquids (ILs) based on choline, the most significant class of green media, have become a more attractive research subject in recent years.^21,22^ These ionic liquids have been extensively employed in an extensive variety of chemical processes due to their abundance, cost-effectiveness, biodegradability, high water solubility, and nontoxicity. Furthermore, Biswal and co-workers discovered^23^ that choline hydroxide can be used as a low-cost organocatalyst for nitrile hydration, indicating the importance of hydrogen bonding in nitrile group activation.

Carbon nanotubes (CNTs) are made of graphite, another allotrope of carbon. All that exists in graphite is a suitable arrangement of carbon atoms in a planar-layered structure. The way that carbon is arranged in graphite is similar to a hexagonal honeycomb lattice. The hollow, cylinder-shaped structure of CNTs is framed by the movement of one or more graphene sheet layers. Depend on the amount of essence of the wall or outer layer. Single-walled carbon nanotubes (SWNTs) and multi-walled carbon nanotubes (MWNTs) are the two most common types of carbon nanotubes (CNTs). Carbon nanotubes have demonstrated considerable limitations in natural and pharmaceutical applications when utilized in aqueous conditions. This issue is resolved through functionalization, which can be achieved through the use of a variety of molecules, absorption, electrostatic contact, or covalent bonding.^24^ The oxidation technique and covalent reactions can be used to develop the function of carbon nanotubes. Oxidizing chemicals such as concentrated sulfuric acid, oxygen air, nitric acid, and aqueous hydrogen peroxide are used in the oxidation process.^25^ A multitude of applications can be utilized across several areas, including sensors, energy, nanomedicine, and environmental research.^26^ Utilizing carbon nanotubes has gained significant popularity in the field of catalysis, among other applications. Carbon nanotubes (CNTs) have been demonstrated to function as catalytic supports or, more frequently, as actual catalysts in numerous studies.^27^

Using CNTs in catalysis based on their combination with inorganic phases is a more common application. The final component is known as a hybrid or composite.^28^ These materials can be generally categorized into three main groups: CNTs and molecular metal catalysts; CNTs and metal nanoparticles (MN/CNTs); and CNTs and transition metal oxides (MO_*x*_/CNTs). Both *in situ* and *ex situ* techniques are used in synthetic strategies. A variety of metal complexes can be attached to carboxylic groups, which are created during CNT oxidation. Wilkinson's catalyst, which included the RhCl(PPh)_3_ complex, was successfully evaluated in the hydrogenation of cyclohexene at room temperature.^29^ It was attached to o-SWCNTs using the carboxyl acid moieties. In recent years, CNT research has focused on heteroatom doping as an alternate functionalization technique.^30^ Applications of graphitic carbons, such as catalysts,^31^ energy conversion/storage devices,^30,32^ and flexible electronics,^33,34^ may benefit from the modified electrical properties^35^ and surface reactivity of N-doped sites. Combining electro-rich nitrogen (N), sulfur (S), and phosphorus (P)-doped carbon nanotubes (CNTs) with other functional elements may maximize performance by promoting cooperative relationships.^36–39^

In this study, *N*-arylamides were synthesized in an environmentally friendly method using a magnetic heterogeneous catalyst made of nitrogen-doped carbon nanotubes (NCNT@Fe_3_O_4_–Cu_2_O). This catalyst has the ability to catalyze the coupling reaction of amides produced by nitrile hydrolysis with aryl halides. Therefore, it is possible to efficiently synthesize *N*-aryl amides by combining two copper-catalyzed procedures. In this research, we continue our interest in green synthesis by using ChOH/Gly to synthesize *N*-arylamides. This chemical operated as a solvent and a base, allowing it to neutralize the acid of hydrogen halide (HX) without the use of another base.

**Scheme 1:**
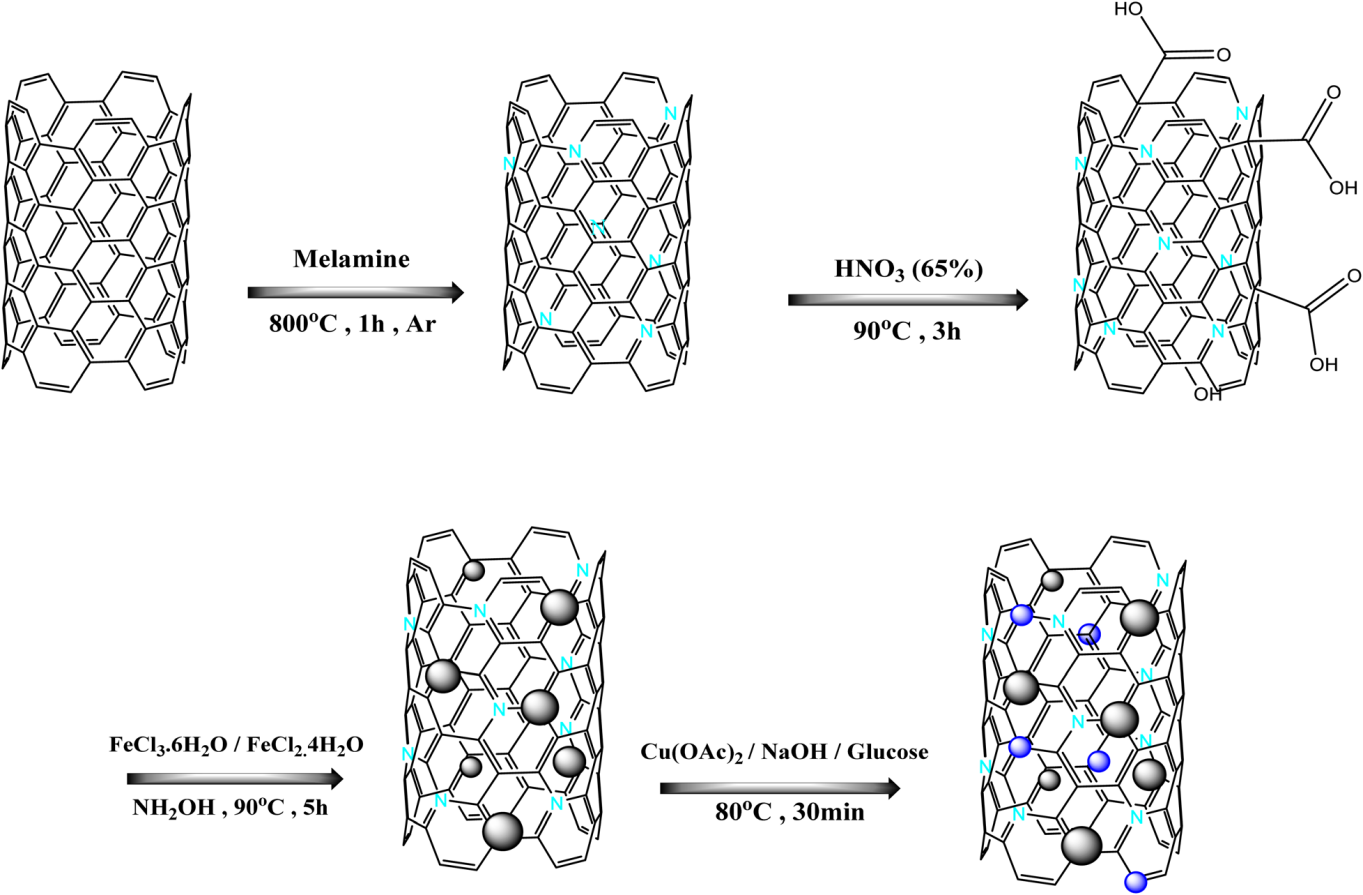
Schematic diagram of N-CNT/Fe_3_O_4_–Cu_2_O.

A synthesis procedure of the catalyst is clearly shown in Scheme 1. The catalyst is characterized by FT-IR, XRD, SEM, EDAX, TEM, TGA, BET, and VSM analyses.

## 2 Experimental

### 2.1. Materials and methods

The required materials were obtained from Merck and employed without further purification. X-ray diffraction (XRD) data were acquired at ambient temperature using a Philips X-pert 1710 with monochromated Cu Kα radiation across the 2*θ* range of 8–80°. Infrared spectra (IR) within the 400–4000 cm^−1^ range were acquired using KBr pellets on a Nicolet IR100 spectrometer. The morphology and dimensions of the particles were evaluated using scanning electron microscopy on a TESCAN MIRA III FE-SEM. EDAX is a method employed to analyze the elemental constituents of catalysts. Transmission electron microscopy (TEM) was conducted at 120 kV using a Philips CM 120. In order to perform the analysis, 10 mg of the sample is dispersed in the ethanol solvent.

Brunauer–Emmett–Teller (BET) surface area of the nanocatalyst was determined by measuring nitrogen gas physisorption on the BELSORP MINI II instrument at 77 K. Inductively coupled plasma (ICP) analysis was performed following acid digestion with a Varlen VISTA-PRO ICP-OE spectrometer. The 1H NMR spectra were acquired utilizing the Bruker DRX-500 Avance at a frequency of 500 MHz, mass spectra were recorded on a Finnigan-MATT 8430 mass spectrometer operating at an ionization potential of 70 eV.

### 2.2. Preparation of nitrogen-doped nanocarbons (NCNT)

Carbon nanotubes and melamine were measured in a one-to-five ratio, combined in a mortar, and thoroughly ground to produce a uniform gray powder. The resultant powder was transferred to a porcelain crucible and subjected to argon gas for 1 h at 800 °C, with a heating rate of 5 °C min^−1^. The product generated at the preceding stage (N-CNT) was rinsed with a dilute hydrochloric acid solution to remove free nitrogen from the material. The composition was afterward rinsed multiple times with deionized water to achieve pH neutralization. Carbon nanotubes were ultimately isolated *via* centrifugation and subsequently dried in an oven.

### 2.3. Activation of nitrogen-doped nanocarbons (NCNT-COOH)

Initially, 1 g of nitrogen-doped carbon nanotubes was measured and put into a balloon, subsequently followed by the addition of 20 mL of 65% nitric acid. In an ultrasonic bath, it was sonicated for 15 to 20 minutes. The resulting mixture was refluxed for 3 hours at 90 °C in an oil bath. The active nitrogen-doped nanotubes, which had been washed multiple times with deionized water, were isolated *via* centrifugation after the specified duration. The functionalized carbon nanotube was oven-dried for 12 h at 100 °C.

### 2.4. Fabrication of magnetic nitrogen-doping carbon nanotubes (N-CNT/Fe_3_O_4_)

5 mmol of iron(iii) chloride hexahydrate (FeCl_3_·6H_2_O) and 10 mmol of iron(ii) tetrahydrochloride (FeCl_2_·4H_2_O) were added to a flask containing 100 mL of DI water and heated and stirred for 1 h at 90 °C. Then, 0.4 g of nitrogenized carbon nanotubes were added to a solution containing 10 mL of 25% ammonium hydroxide in 50 mL of deionized water and dispersed in an ultrasonic bath. Instantly, the two mixtures were transferred to a 250 mL flask and stirred at 90 °C with Ar gas for 5 h. The resultant solid followed multiple washings, was separated by an external magnet, and was then dried in an oven at 50 °C.

### 2.5. Preparation of copper oxide nanoparticles decorated on magnetic nitrogen-doping carbon nanotubes (N-CNT/Fe_3_O_4_–Cu_2_O)

2.8 mmol of copper acetate was dispersed in 30 mL of water. Subsequently, 150 mg of the N-CNT/Fe_3_O_4_ substrate that was made in the preceding step was added to the copper acetate solution, and it was stirred for 12 h at room temperature. Then they overflowed the copper solution and added water to it again. Then the amounts 0.5 g of glucose and 0.5 g of sodium (NaOH) were added to the mixture and placed in an oil bath with a temperature of 80 °C for 30 min. Next, multiple washings were performed on the resultant nanoparticles using a 1 : 1 ethanol/water solution to remove any remaining contaminants. After that, it was dried for 12 h at 70 °C in an oven.

### 2.6. Preparation of choline hydroxide

Choline chloride (10 mmol, 1.39 g) and KOH (20 mmol, 1.12 g) in methanol (20 mL) were put in an oil bath at 60 °C for 10 h under N_2_. Following the reaction mixture's cooling to room temperature, solid KCl was removed by filtering it, and methanol was eliminated by concentrating the solution under reduced pressure.

### 2.7. Preparation of *N*-arylbenzamides

1 mmol (0.103 g) of benzonitrile and 1.2 mmol (0.244 g) of iodoarene were added to a reaction tube with 3 mL of deep eutectic solvent (made from a 1 : 2 combination of choline hydroxide and glycerol that was stirred for an hour at 80 °C). The mixture was followed by mixing with 0.02 g of catalyst. The resulting mixture was stirred in an oil bath at a temperature of 100 °C for 6 h, and after the completion of the reaction, the catalyst was separated from the reaction mixture using an external magnet, and the product was extracted by a mixture of water and ethyl acetate. Excess solvent was evaporated under reduced pressure. The product was purified by column chromatography on silica gel.

## 3 Results and discussion

### 3.1. Catalyst characterization

**Fig. 1:**
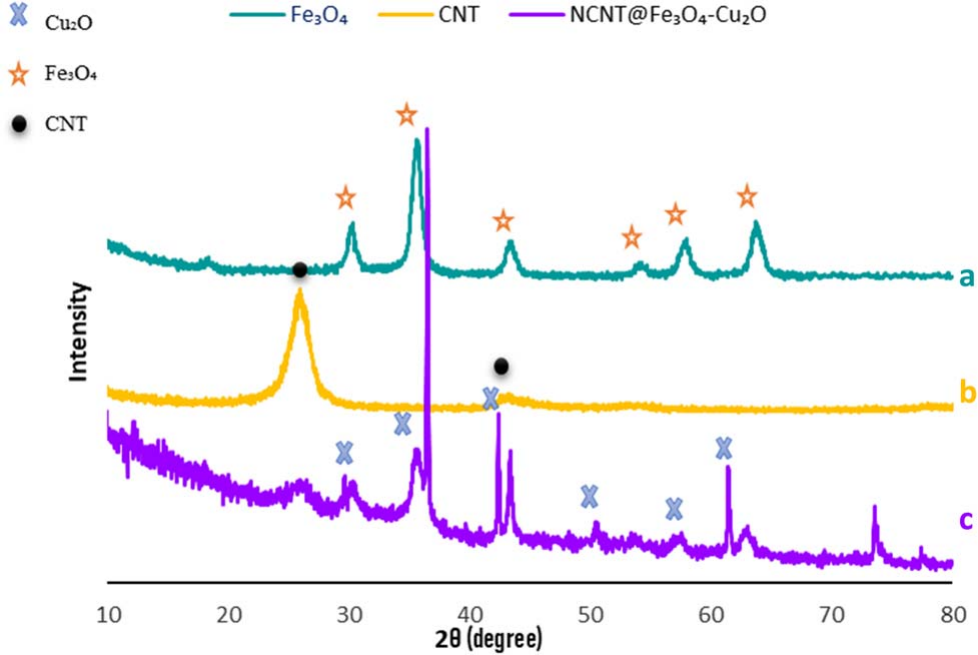
PXRD of Fe_3_O_4_ (a), CNT (b), NCNT@Fe_3_O_4_–Cu_2_O (c).

X-ray diffraction analysis should be carried out in order to determine the catalyst's chemical composition and crystal structure. The X-ray diffraction patterns of the samples are displayed in Fig. 1. According to Fig. 1a, in the spectrum of ferrite oxide, the diffraction peaks observed at the angles 30.86°, 35.60°, 43.15°, 53.81°, 57.19°, and 63.1° correspond to the Bragg plates 220, 311, 400, 422, 511, and 440, respectively, and are completely consistent with the diffraction patterns of magnetite (Fe_3_O_4_) according to JCPDS Card 19-0629,^40^ and no more peaks appear to indicate the presence of contaminants. In the spectrum of nanotubes, the angles (2*θ*), 26.3° and 42.6°, correspond to the 002 and 100 planes,^41^ respectively. The appearance of Fe_3_O_4_ and N-CNT diffraction peaks in the NCNT/Fe_3_O_4_–Cu_2_O composite patterns shows that Fe_3_O_4_ has been successfully loaded onto N-CNTs. In the final spectrum of the copper catalyst (I) fixed on the nitrogen nanotube, there are diffraction peaks at the angles of 29.6°, 36.4°, 42.3°, 61.3°, 73.5°, and 77.3°, according to pages 110, 111, 200, 220, 311, and 222, respectively, showing copper metal oxide^42^ stabilized on magnetic nitrogenized nanosheets. Furthermore, the strength of the Cu_2_O (111) peak is significantly greater than that of the other peaks, which can be attributed to the (111) crystal plane's strong preferred orientation.

**Fig. 2:**
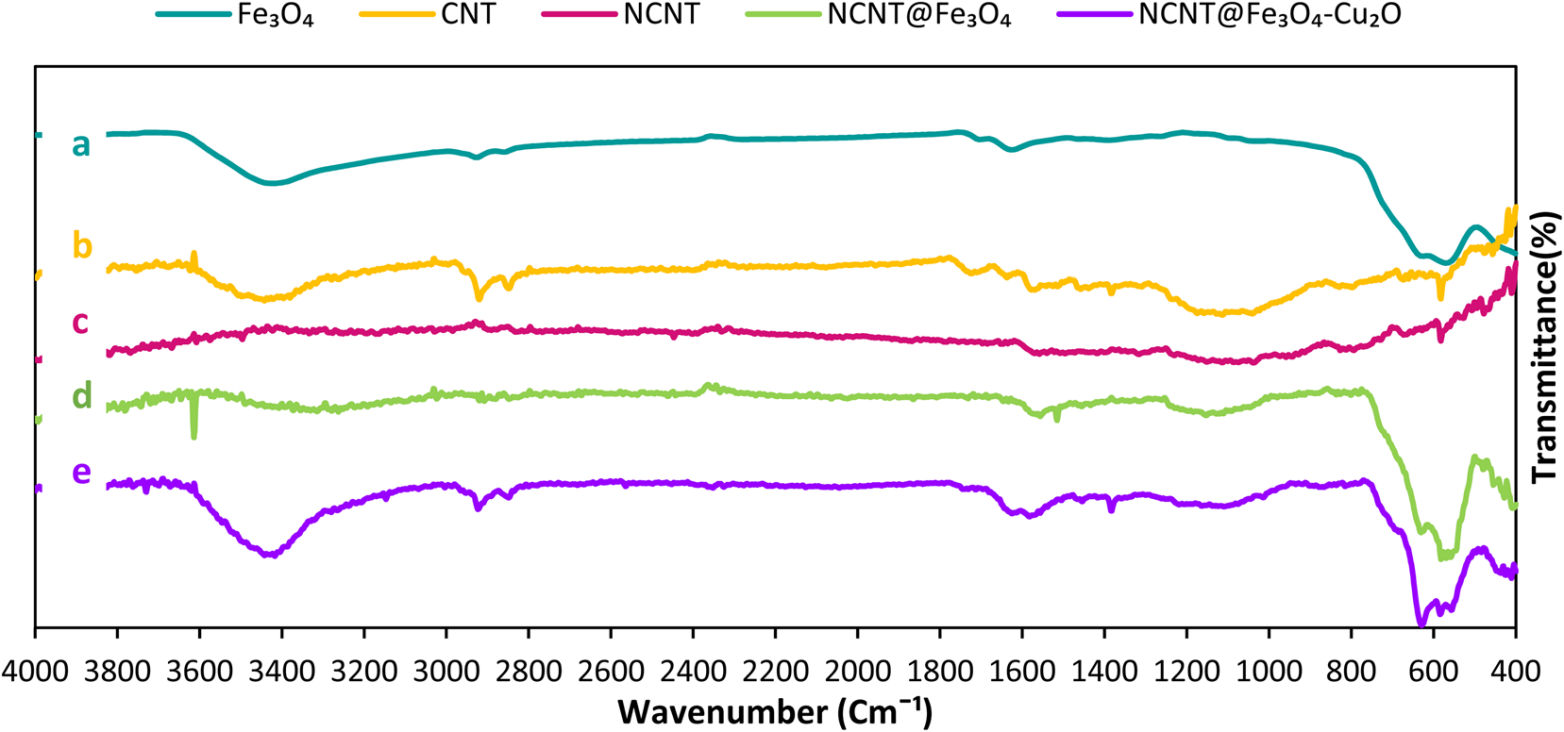
FT-IR of Fe_3_O_4_ (a), CNT (b), NCNT (c), NCNT@Fe_3_O_4_ (d), NCNT@Fe_3_O_4_–Cu_2_O (e).

FT-IR spectroscopy was used to find functional groups to gain insight into changes that happened at each stage of the catalyst production procedure. FT-IR spectra of Fe_3_O_4_, CNT, N-CNT, N-CNT/Fe_3_O_4_, and N-CNT/Fe_3_O_4_–Cu_2_O are depicted in Fig. 2. As illustrated in Fig. 2a, The Fe_3_O_4_ spectrum^43^ was roughly attributed as follows: 3420 cm^−1^ to the O–H stretching vibration; 580 cm^−1^ to the Fe–O band. The FT-IR spectra of CNT show that the bands at 1700 cm^−1^ and in the 3400 cm^−1^ regions correspond to the stretching vibration of the C=O and O–H carboxyl groups in CNT^44^ (Fig. 2b). The presence of cylinder-like carbon structures was indicated by the band seen in the CNT's FTIR spectra at 1400 cm^−1^ and 1600 cm^−1^. The N-CNT spectrum was roughly attributed to what follows: 3200 cm^−1^ to N–H stretching vibrations; 1530 cm^−1^ to the N–H bending vibration; 1400 cm^−1^ to the C–N stretching vibration; these observations confirm the successful formation of the N-CNT. The N-CNT/Fe_3_O_4_ FT-IR spectra (Fig. 2d) revealed the absorption bands of both N-CNT and Fe_3_O_4_, confirming Fe_3_O_4_'s successful attachment to the N-CNT surface. Fig. 2e displays the N-CNT/Fe_3_O_4_–Cu_2_O FT-IR spectrum. Fig. 2e demonstrates that, in addition to the N-CNT/Fe_3_O_4_ peaks present in the final composite, an extra peak at 553 cm^−1^ is ascribed to Cu–O bonds, which constitute the copper oxide structure.

**Fig. 3:**
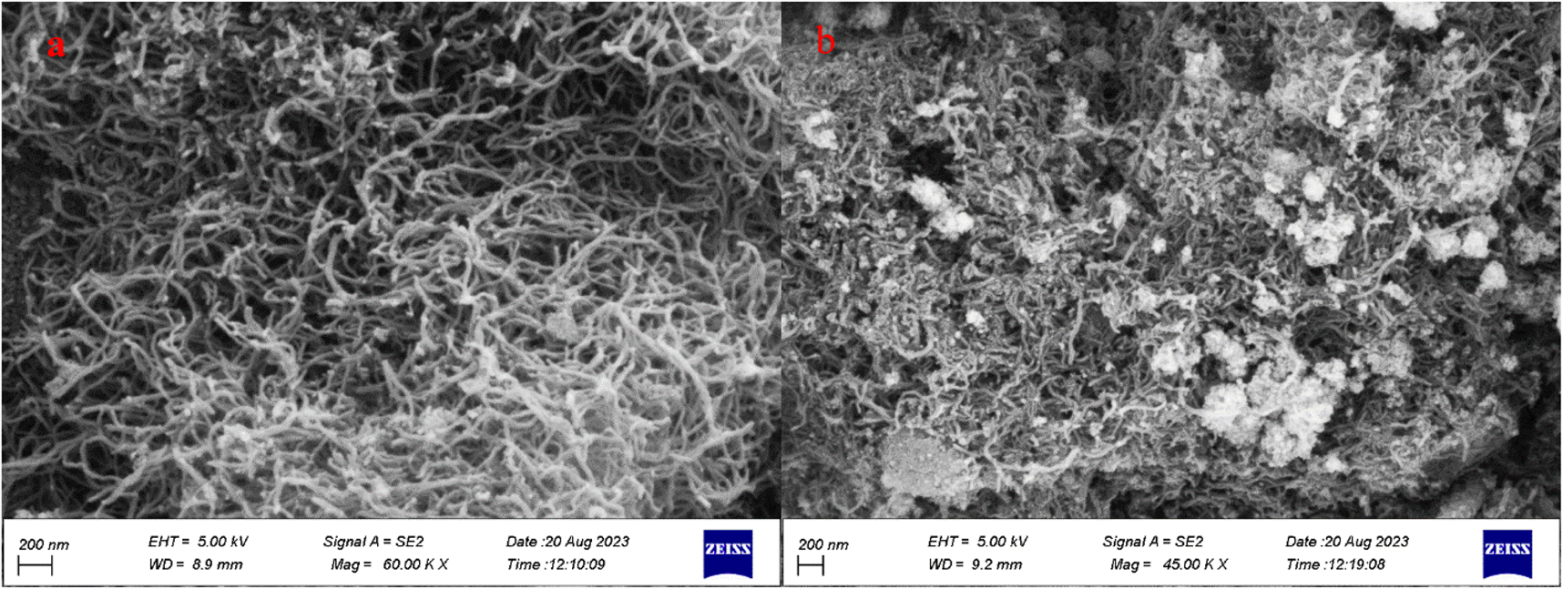
FE-SEM of CNT (a), NCNT@Fe_3_O_4_–Cu_2_O (b).

Scanning electron microscopy (SEM) was applied to evaluate the size and surface morphology (Fig. 3). Fig. 3a shows the FESEM images of the purified CNTs, and Fig. 3b shows the FESEM images of the N-CNT/Fe_3_O_4_–Cu_2_O that indicated spherical nanoparticles of iron and copper(i) oxide grew properly onto the surface of N-CNT.

**Fig. 4:**
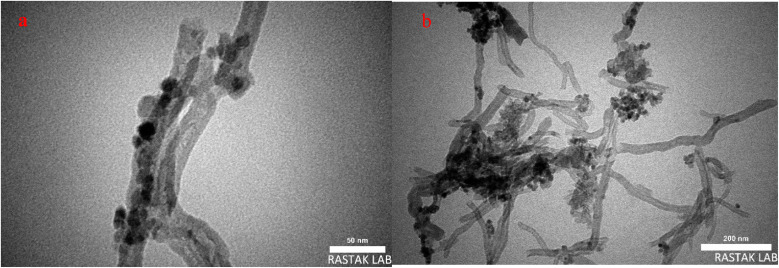
TEM of NCNT@Fe_3_O_4_–Cu_2_O (a and b).

TEM spectroscopy is used to evaluate the morphology of nanoparticles. The TEM images of N-CNT/Fe_3_O_4_–Cu_2_O (Fig. 4a, b) show that the CNTs are covered with nearly spherical nanoparticles of Cu_2_O and Fe_3_O_4_, confirming the successful immobilization of the MNPs and Cu_2_O on the N-CNT surface.

**Fig. 5:**
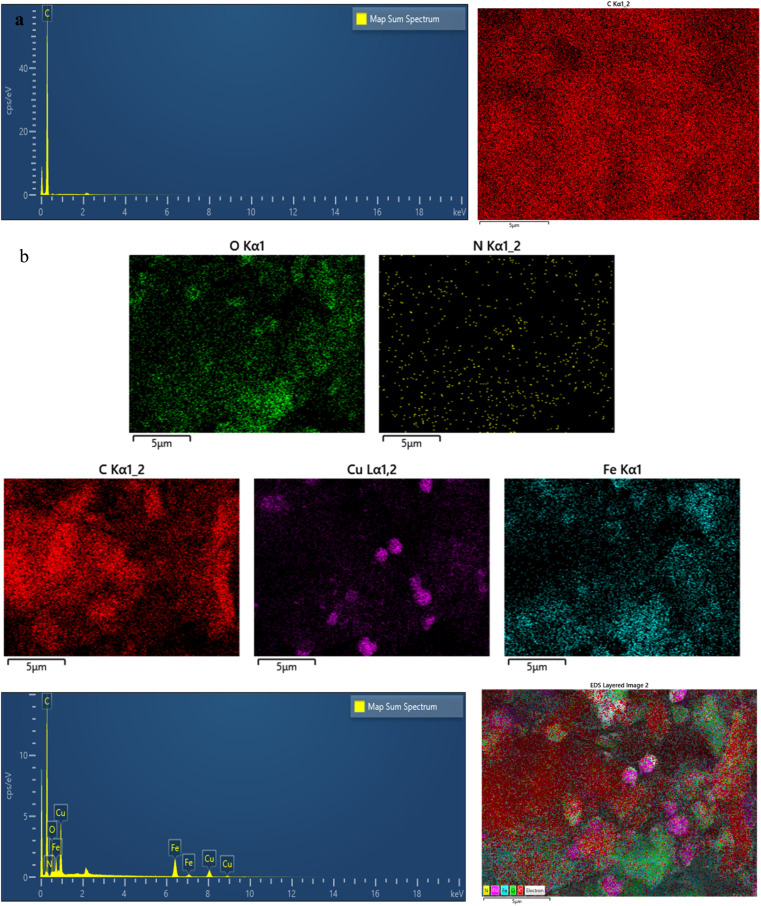
EDAX analysis of CNT (a), NCNT@Fe_3_O_4_–Cu_2_O (b).

In Fig. 5(a and b), the X-ray energy diffraction (EDAX) of two samples, pure carbon nanotube in Fig. 5a, and Cu_2_O and Fe_3_O_4_ stabilized on N-doped nanotubes (N-CNT/Fe_3_O_4_–Cu_2_O) in Fig. 5b, are given, which shows that the desired nanoparticles have been successfully attached to the carbon substrate.

**Fig. 6:**
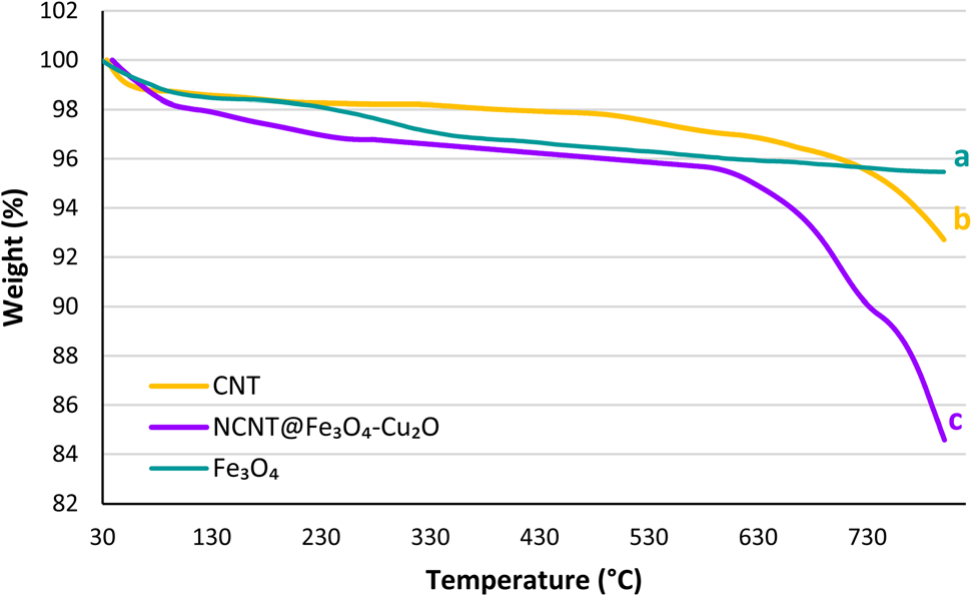
TGA curves of Fe_3_O_4_ (a), CNT (b), NCNT@Fe_3_O_4_–Cu_2_O (c).

The thermogravimetric analysis (TGA) was utilized to measure the thermal stability of nanocomposites. Thermal decomposition analysis reveals changes in sample weight as a function of temperature, which can provide significant information concerning the physical and chemical changes of the nanocomposite at various temperatures. TGA curves of Fe_3_O_4_, CNT, and N-CNT/Fe_3_O_4_–Cu_2_O are presented in Fig. 6(a–c). The TGA curve of pure Fe_3_O_4_ (Fig. 6a) exhibited little change in terms of weight loss at a temperature below 200 °C, which can be mainly attributed to water desorption and the elimination of hydroxyl groups. For N-CNT/Fe_3_O_4_–Cu_2_O, two major mass loss steps were observed (Fig. 6c). The initial weight loss of the catalyst is noticed at temperatures below 200 °C, which can be connected to the volatilization of interlayer and absorbed water with a weight loss of about 3.0%. The resulting nanocomposite is relatively stable between 200 and 600 °C; as the temperature rises, the organic groups on the structure are destroyed. Compared to pure carbon nanotubes, the composite structure is less stable at high temperatures; up to 800 °C, roughly 12% of the structure is degraded.

**Fig. 7:**
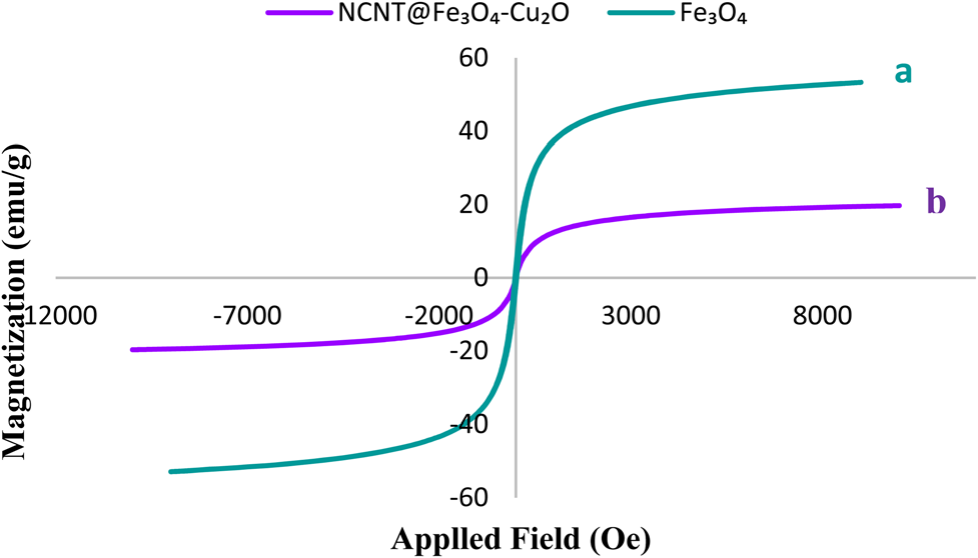
VSM of Fe_3_O_4_ (a), NCNT@Fe_3_O_4_–Cu_2_O (b).

Vibrating sample magnetometer (VSM) analysis was used to examine the magnetic behavior of the N-CNT/Fe_3_O_4_–Cu_2_O catalyst. Fig. 7 illustrates the saturation magnetization of N-CNT/Fe_3_O_4_–Cu_2_O, which is around 20 emu g^−1^. The presence of non-magnetic species surrounding Fe_3_O_4_ is the reason why the value of *M*_s_ for the composite was lower than that of pure Fe_3_O_4_ (40 emu. g^−1^). But because of its single-curve shape, the structure still has superparamagnetic properties.

**Fig. 8:**
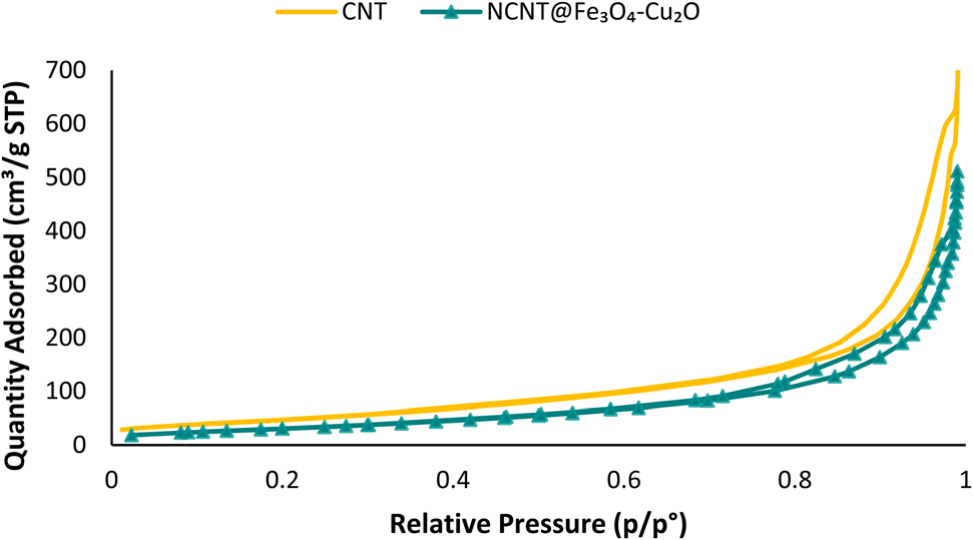
BET of CNT (yellow), NCNT@Fe_3_O_4_–Cu_2_O (green).

Browner–Emmett–Teller (BET) analysis is a physical method used to determine the absorption or desorption of gas molecules on surfaces or inside cavities. The size of the pores determines the volume of gas absorbed. Based on this, it is possible to determine the approximate size and shape of the pores by monitoring the amount of gas entering and leaving the pores. Fig. 8 depicts the type V isotherms in the adsorption–desorption curves of the CNT and N-CNT/Fe_3_O_4_–Cu_2_O, which are typical of mesoporous materials. According to calculations, the BET surface areas of CNT and N-CNT/Fe_3_O_4_–Cu_2_O were 173.93 and 117.73 m^2^ g^−1^, respectively. As shown in Fig. 8, when N-CNT and Fe_3_O_4_ are combined, the nanocomposite's specific surface area decreases.

### 3.2. Application of NCNT@Fe_3_O_4_–Cu_2_O catalyst in reaction

Following the fabrication and characterization of the nanocatalyst, its efficiency in the production of *N*-arylbenzamides was evaluated (Table 1). The investigation started with a model reaction involving iodobenzene (**1a**) and benzonitrile (**2a**), and a number of significant factors were studied, including the solvent, base, reaction temperature, time, type (Table 1), and amounts of catalysts. We initially focused on the catalytic efficiency of the NCNT@Fe_3_O_4_–Cu_2_O catalyst, which includes several heterogeneous catalysts (Table 1, entries 1–5) in the synthesis of *N*-arylbenzamides. The synthesized CNT, NCNT, NCNT@Fe_3_O_4_, and NCNT@Fe_3_O_4_–CuO nanoparticles were evaluated, resulting in product yields of 0%, 0%, 15%, and 55%, respectively. Under the same conditions, the synthesized NCNT@Fe_3_O_4_–Cu_2_O nanocatalyst (Table 1, entry 4) was suitable for this reaction due to its outstanding reaction efficiency and yield, which was 91%. Furthermore, catalysts such as CuI and CuBr appeared to be less effective (Table 1, entries 16 and 17). We examined multiple solvents to identify the most suitable one for synthesizing *N*-arylbenzamides (Table 1, entries 6–12). The most suitable solvent among those listed was ChOH/Gly, as this deep eutectic solvent functions as both a base and a solvent, eliminating the need for external bases and solvents. The selection of the base was shown to be critical to this reaction. ChOH yielded the most favorable findings among all evaluated bases (Cs_2_CO_3_, KOH, K_2_CO_3_, and ChOH) with a 62% yield (Table 1, entries 10, and 13–15). Under optimal conditions, we also concentrate on optimizing the catalyst quantity, which ranges from 10 mg to 30 mg (Table 1, entries 18–20). The highest yield findings are obtained with 20 mg of NCNT@Fe_3_O_4_–Cu_2_O, and the yields are not significantly affected by further increasing the catalyst quantity (Table 1, entry 18). Then, the effect of temperature on the reaction was investigated. An additional enhancement in product yield to 91% was attained by increasing the reaction temperature to 100 °C (Table 1, entries 23, 24, and 4). Furthermore, investigations conducted at temperatures above 100 °C detected no enhancement in reaction yield (Table 1, entries 21 and 22). In the end, the reaction was timed at several intervals, and the results showed that 5 hours was the ideal time for both energy consumption and product production (Table 1, entries 16 and 17).

**Table 1:** Optimization of the *N*-arylbenzamides synthesis^a^

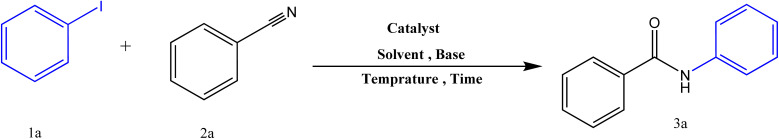						
Entry	Catalyst (mg)	Solvent	Base	Temperature (^o^C)	Time (h)	Yield^b^ (%)
1	CNT (20)	ChOH/Gly	—	100	10	n.r.
2	NCNT (20)	ChOH/Gly	—	100	10	n.r.
3	NCNT@Fe_3_O_4_ (20)	ChOH/Gly	—	100	10	15
**4**	**NCNT@Fe**_**3**_ **O**_**4**_ **–Cu**_**2**_ **O (20)**	**ChOH/Gly**	**—**	**100**	**5**	**91**
5	NCNT@Fe_3_O_4_–CuO (20)	ChOH/Gly	—	100	5	55
6	NCNT@Fe_3_O_4_–Cu_2_O (20)	ChOH/Gly	K_2_CO_3_	100	5	87
7	NCNT@Fe_3_O_4_–Cu_2_O (20)	Glycerol	K_2_CO_3_	100	5	Trace
8	NCNT@Fe_3_O_4_–Cu_2_O (20)	H_2_O	K_2_CO_3_	100	5	12
9	NCNT@Fe_3_O_4_–Cu_2_O (20)	1,4-Dioxane	K_2_CO_3_	100	5	Trace
10	NCNT@Fe_3_O_4_–Cu_2_O (20)	DMF	K_2_CO_3_	100	5	55
11	NCNT@Fe_3_O_4_–Cu_2_O (20)	Toluene	K_2_CO_3_	100	5	45
12	NCNT@Fe_3_O_4_–Cu_2_O (20)	DMSO	K_2_CO_3_	100	5	Trace
13	NCNT@Fe_3_O_4_–Cu_2_O (20)	—	ChOH	100	5	62
14	NCNT@Fe_3_O_4_–CuO (20)	DMF	Cs_2_CO_3_	100	5	42
15	NCNT@Fe_3_O_4_–CuO (20)	DMF	KOH	100	5	28
16	CuI (10 mol%)	ChOH/Gly	—	100	8	13
17	CuBr (10 mol%)	ChOH/Gly	—	100	8	n.r.
18	NCNT@Fe_3_O_4_–Cu_2_O (30)	ChOH/Gly	—	100	5	89
19	NCNT@Fe_3_O_4_–Cu_2_O (15)	ChOH/Gly	—	100	5	78
20	NCNT@Fe_3_O_4_–Cu_2_O (10)	ChOH/Gly	—	100	5	61
21	NCNT@Fe_3_O_4_–Cu_2_O (20)	ChOH/Gly	—	110	5	89
22	NCNT@Fe_3_O_4_–Cu_2_O (20)	ChOH/Gly	—	120	5	85
23	NCNT@Fe_3_O_4_–Cu_2_O (20)	ChOH/Gly	—	90	5	67
24	NCNT@Fe_3_O_4_–Cu_2_O (20)	ChOH/Gly	—	80	5	52
25	NCNT@Fe_3_O_4_–Cu_2_O (20)	ChOH/Gly	—	100	2	39
26	NCNT@Fe_3_O_4_–Cu_2_O (20)	ChOH/Gly	—	100	4	69
27	NCNT@Fe_3_O_4_–Cu_2_O (20)	ChOH/Gly	—	100	6	92

a Reaction conditions: iodobenzene (1.2 mmol), benzonitrile (1 mmol), solvent (3 mL), catalyst (*x* mg), temperature, time.

b Isolated yields.

The scope of the amination reaction of aryl halides with nitriles was expanded to a variety of substituted aryl iodides and benzonitriles. The yield of the final product diminishes in the presence of strong electron-withdrawing groups, such as nitro and fluoride, as substituents on both benzonitrile and iodobenzene reagents (**3f**, **3g**, **3m**). In the presence of electron-donating groups such as methyl and methoxy, a good yield of the desired product is obtained, but the presence of these groups as substituents on benzonitrile and iodobenzene reagents does not have an increasing effect on the yield (**3c**, **3j**, **3l**). Because of the steric hindrance effect at the *ortho*-position, the yields of the corresponding amide products were reduced when benzonitriles and aryl iodides containing functional groups at the ortho position were converted (**3e**, **3k**, **3m**).

When halogenated substituents are present, the high yield of the products is also preserved (**3d**, **3e**, **3h**, **3i**). The benzamide product is primarily produced by the process of iodine replacement, which is consistent with the fact that iodine has a higher leaving power than bromine and chlorine when it comes to aryl iodides containing halogen functional groups such as chlorine and bromine. Compared to the other halogens, aryl iodides exhibited higher levels of reactivity. The reaction mixture was found to have a low yield of certain aryl halides, particularly aryl bromides and chlorides. Further analysis revealed that neither an increase in temperature nor an extension of the reaction time improved the yields of certain aryl halides (**3b**). In order to investigate the influence that aliphatic nitriles have on this transformation, iodobenzene (**1a**) was utilized as a partner in the reaction with a number of other nitriles. According to the results presented in Table 2, aliphatic nitriles, like aryl nitriles, are capable of producing the desired products with an excellent yield (**3n**, **3o**).

**Table 2:** Synthesis of *N*-arylbenzamides by NCNT@Fe_3_O_4_–Cu_2_O^a^

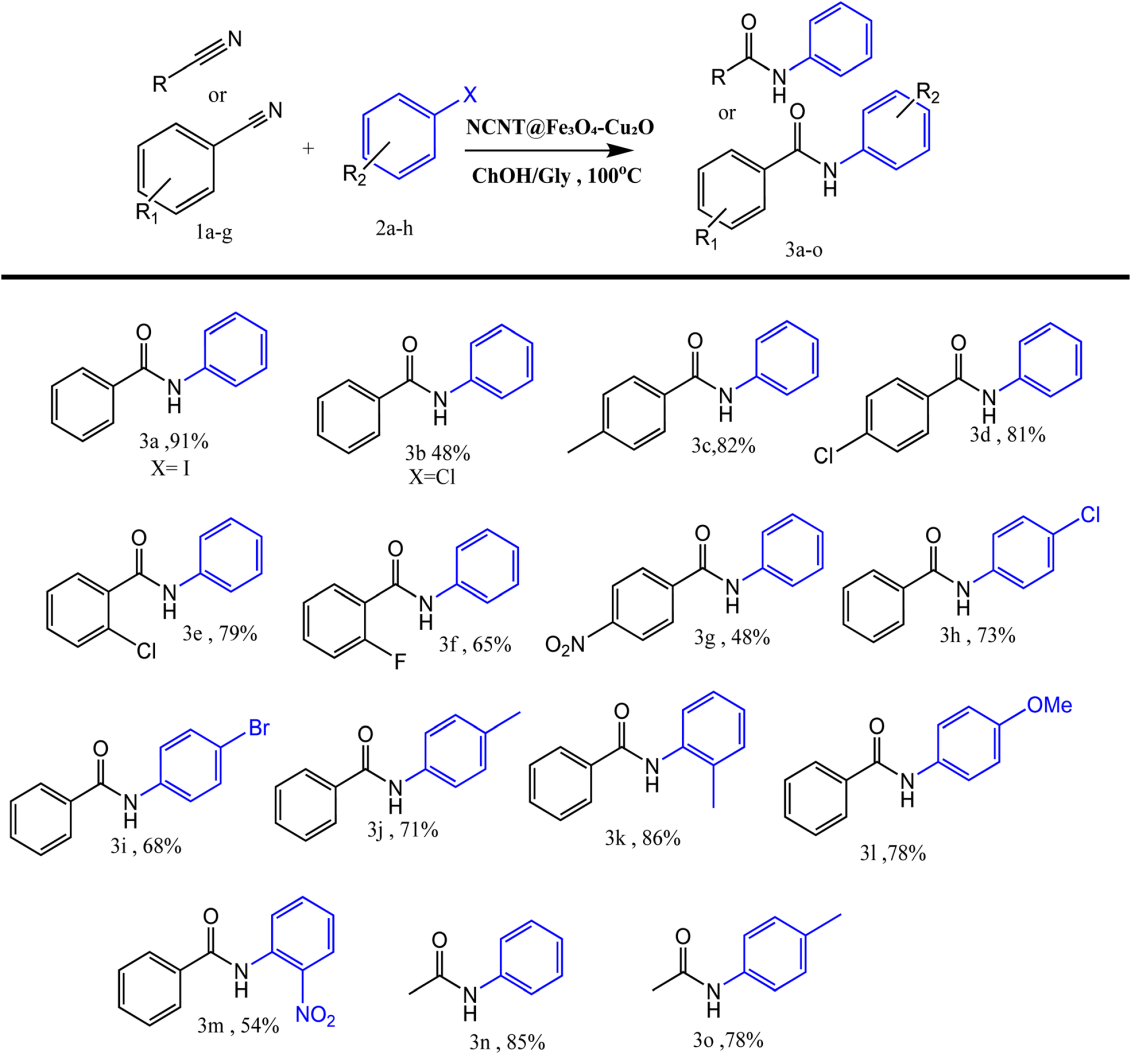

a Reaction conditions: aryl halide (1.2 mmol), benzonitrile (1 mmol), ChOH/Gly (3 mL), N-CNT@ Fe_3_O_4_–Cu_2_O (20 mg), 100 °C, 6 h.

Table 3 indicates a comparison between the current procedure and previous reports. Previous studies indicate that the reactions were conducted at elevated temperatures for a longer time utilizing organic or inorganic bases, phosphorus ligands, and gaseous carbon monoxide, alongside homogeneous or heterogeneous catalysts that created challenges for product separation, requiring centrifugation. This study employed an economical and heterogeneous catalyst to synthesize amides from benzonitrile and iodobenzene utilizing a deep eutectic solvent (ChOH: glycerol). Employing a deep eutectic with both basic and aqueous properties obviates the requirement for an external base and extra solvent. Another advantage of this reaction is the utilization of a heterogeneous catalyst system characterized by excellent efficiency and stability, allowing for straightforward recovery from the reaction mixture through the application of an external field. The current work presented a safe, user-friendly, and appropriate alternative for the synthesis of amides and esters with elevated yields.

**Table 3:** Comparison of different types of *N*-arylbenzamides synthesis

Entry	Catalyst	Reaction condition	Yield (%)	References
1	CuI	Iodobenzene (102.0 mg, 0.5 mmol), benzonitrile (2.0 mL), CuI (9.5 mg, 0.05 mmol), DMEDA (8.0 mL, 0.075 mmol), KOH (42 mg, 0.75 mmol), Cs_2_CO_3_ (81.5 mg, 0.25 mmol), H_2_O (76.5 μL, 4.25 mmol), Ar, 15 h at 100 °C	92	45
2	CuSO_4_·5H_2_O	Iodobenzene (100 mg, 0.49 mmol), benzaldoxime (4 equiv.) in *o*-xylene (1 mL), CuSO_4_·5H_2_O (10 mol%), K_2_CO_3_ (5 equiv.), and DMEDA (30 mol%), 130 °C for 12 h	82	46
3	CuI	CuI (9.5 mg, 0.05 mmol), K_2_CO_3_ (138.2 mg, 1.0 mmol), toluene (2.0 mL), aryl monohalides (0.5 mmol), nitrile (1.0 mmol), DMEDA (8.0 μL, 0.075 mmol), acetaldoxime (92.0 μL, 1.5 mmol), Ar, 120 °C for 15 h	94	47
4	SILP-Pd	Aniline (0.5 mmol), iodobenzene (0.25 mmol), Et_3_N (0.25 mmol), DMF (1 mL), catalyst Pd, CO (30 bar), 100 °C, 8 h	92	48
5	N-CNT/Fe_3_O_4_–Cu_2_O	Benzonitrile (1 mmol, 0.103 g), iodobenzene (1.2 mmol, 0.244 g), ChOH: Glycerol (1 : 2) (3 mL), 0.02 g of catalyst, 100 °C for 6 h	91	This work

### 3.3. Mechanism

**Scheme 2:**
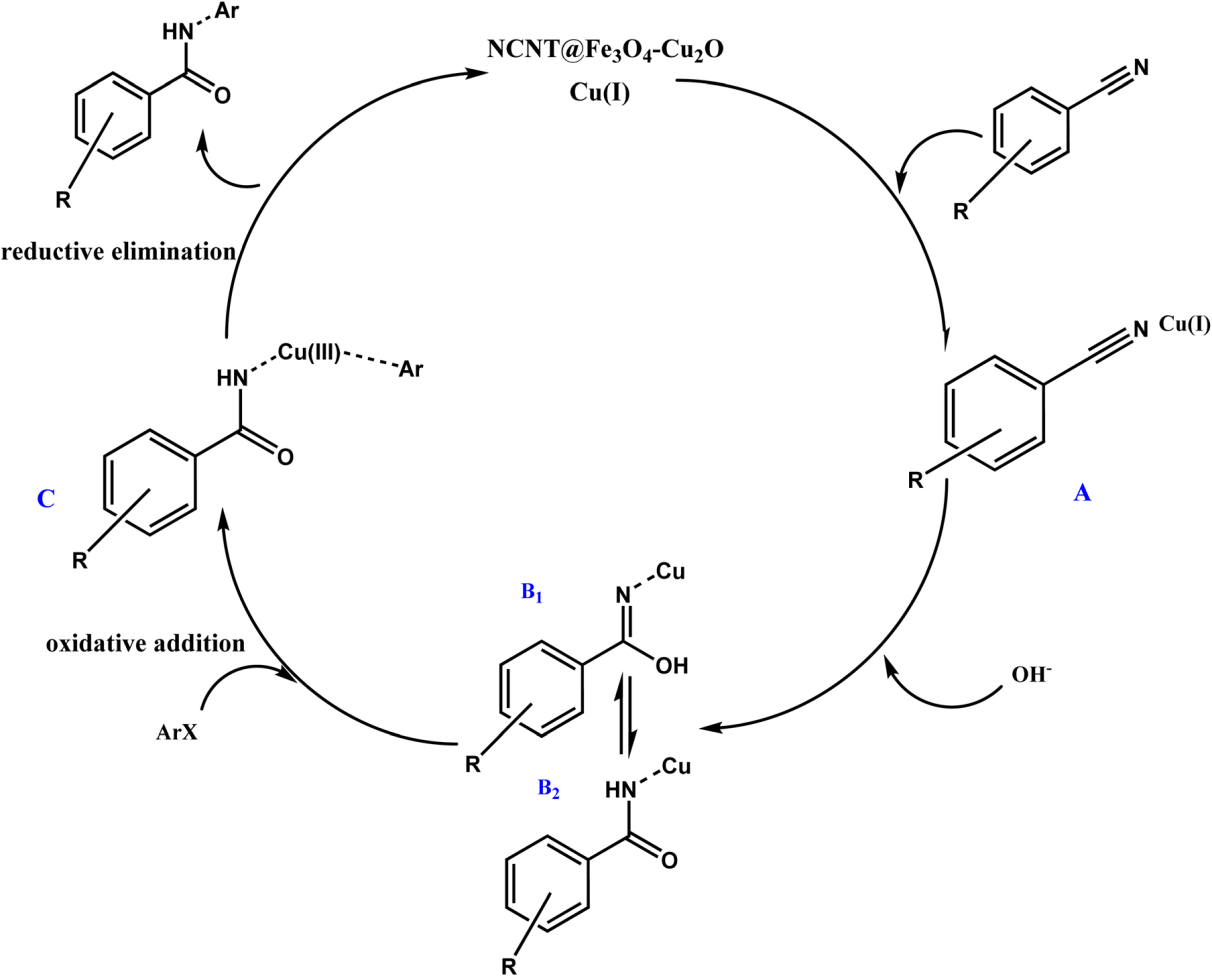
Mechanism of the *N*-arylbenzamides synthesis.

The mechanism of this reaction is depicted in Scheme 2. Benzonitrile is initially coordinated with copper(i) to create complex A. Then, benzonitrile is hydrolyzed by OH^−^ of choline hydroxide and forms benzamide B2 through tautomerism. Benzamide undergoes an oxidative addition reaction with aryl halide in the presence of a copper catalyst, resulting in the formation of product C. Ultimately, in the reductive elimination reaction and the reduction of copper(iii) to copper(i), the final product is generated, and copper returns the catalytic cycle of the reaction.

### 3.4. Reusability of the NCNT@Fe_3_O_4_–Cu_2_O catalyst

**Fig. 9:**
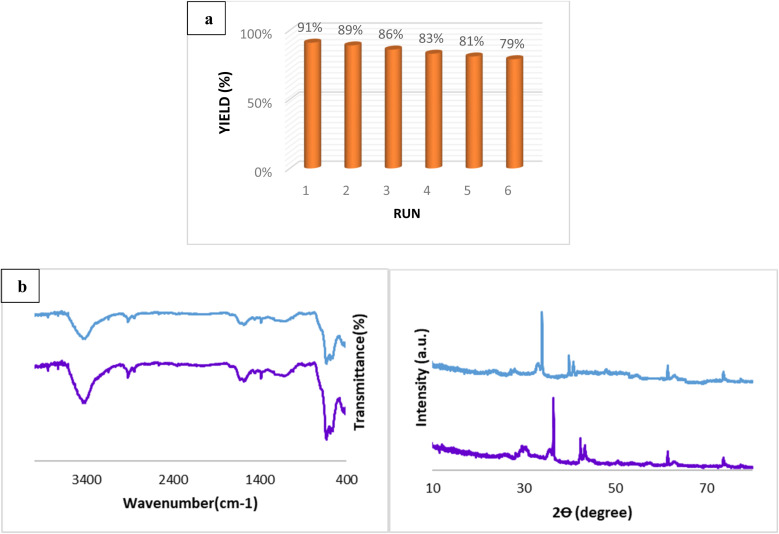
(a) catalyst reusability, (b) FT-IR spectrum, and XRD pattern of the recovered N-CNT/Fe_3_O_4_–Cu_2_O photocatalyst after the 6th run.

Recyclability and reusability potential are two significant advantages of heterogeneous catalysts over homogeneous catalysts. To investigate the catalyst's reusability, we conducted the reaction of iodobenzene with benzonitrile using the NCNT@Fe_3_O_4_–Cu_2_O catalyst as the model reaction. After each cycle, the product was extracted with ethyl acetate, and the NCNT@Fe_3_O_4_–Cu_2_O catalyst was simply separated with an external magnetic bar. The recovered catalyst was washed multiple times with water and methanol to eliminate any organic material before being dried and being used for the next run under the same conditions. The NCNT@Fe_3_O_4_–Cu_2_O catalyst was successfully used in six runs with no significant loss of catalyst activity, as shown in Fig. 9. To investigate structural changes, the recovered catalyst from the aqueous phase in the last run was analyzed using XRD and FT-IR. The results revealed that the FT-IR spectrum and XRD patterns obtained from the recovered catalyst were quite consistent with the fresh catalyst, confirming strong catalytic stability under reaction conditions. The ICP-AES evaluation of the aqueous phase in the sixth run and the final organic phase revealed that the leached copper species was less than the detectable limit, confirming the strong interaction between Cu nanoparticles and support.

## 4 Conclusion

In summary, we developed a one-step protocol for the direct conversion of nitriles into *N*-arylated amides in the presence of a heterogeneous copper(i) catalyst. We created an amination reaction using aryl halides and nitriles. The utilization of nitriles as nitrogen nucleophiles can facilitate the synthesis of *N*-arylamides more efficiently than that derived from amides by hydrolysis. ChOH/Gly was employed in the synthesis of *N*-arylamides in the current study as part of our ongoing research interest in green synthesis. This compound served a dual function as a solvent and as a base to scavenge the acid of hydrogen halide (HX) without the need for an additional base. This compound also functions as a solvent to hydrolyze nitrile and transform it into amide. This study utilized a magnetic heterogeneous catalyst composed of nitrogen-doped carbon nanotubes (NCNT@Fe_3_O_4_–Cu_2_O) to synthesize *N*-arylamides under eco-friendly conditions. Excellent to good yields of products were achieved in the presence of the catalyst. A special advantage of the catalyst is the ability to recover it by applying an external magnetic field.

## Data availability

The data that supports the findings of this study are available in the ESI† of this article.

## Conflicts of interest

There are no conflicts of interest to declare.

## Supplementary Material

RA-015-D5RA00849B-s001
