# Supplementary material for: Magnetic N-doped CNT stabilized Cu2O as a catalyst for N-arylation of nitriles and aryl halides in a biocompatible deep eutectic solvent
Source: RSC Adv. 2025 Mar 18;15(11):8195–206. doi: 10.1039/d5ra00849b (PMC11915213; doi:10.1039/d5ra00849b)
Supplement: RA-015-D5RA00849B-s001 [file RA-015-D5RA00849B-s001.pdf]

Supplementary Information (SI) for RSC Advances.
This journal is © The Royal Society of Chemistry 2025

Magnetic N-doped CNTs Stabilized Cu_2O as Catalyst for N-arylation of Nitriles and Aryl Halides in a Biocompatible Deep Eutectic Solvent

Mohadeseh Alizadeh[a], Arefe Salamatmanesh[a], Masoumeh Jadidi Nejad[*b], Akbar Heydari[*a]

[a]*Chemistry Department, Tarbiat Modares University, P.O. Box 14155-4838, Tehran, Iran.*

[b]*Department of Chemistry, Isfahan University of Technology, Isfahan, 84156-83111, Iran.*

[*b] *E-mail: m.jadidinejad@iut.ac.ir; Tel: +98-31-33913261*

[*a] *E-mail: heydar_a@modares.ac.ir; Fax: +98-21-82883455; Tel: +98-21-82883444*

Table of contents:

Characterization Data for the products S2

^{1}H NMR and ^{13}CNMR spectra of products S6

References S20

Characterization data of products:

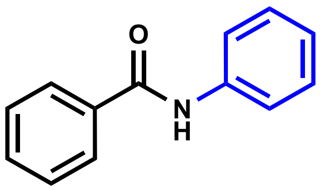

3a ,91% X= I
3b 48% X=Cl

N-phenylbenzamide (3a-3b)[1]: Ligh brown solid; (3a: 91%- 3b: 48%) isolated yield; Mp: 157-160 °C; ^{1}H NMR (500 MHz, DMSO-d$_6$): δ (ppm) = 10.25 (s, 1H), 7.96 (d, J=6.5Hz, 2H), 7.79 (d, J=7.9Hz, 2H), 7.61-7.55 (m, 1H), 7.52 (t, J=6.9 Hz, 2H), 7.35 (t, J=7.2 Hz, 2H), 7.1 (t, J=7.3 Hz, 1H); ^{13}C NMR (101 MHz, CDCl$_3$) δ 165.8 (CO amide), 137.8 (C), 135.5 (C), 131.6 (CH), 129.2 (CH), 128.6 (CH), 127.2 (CH), 124.5 (CH), 120.5 (CH); IR (KBr): ν (cm^{-1})= 3343, 3050, 2921, 1654, 1598, 1438, 1275, 749, 689; MS (EI, 70eV): m/z: 197[M]$^+$.

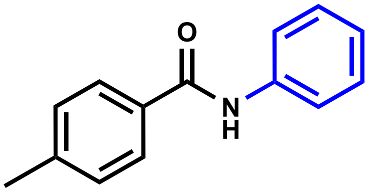

3c,82%

4-methyl-N-phenylbenzamide (3c): white solid 82% isolated yield; Mp: 146-148°C; ^{1}H-NMR (500 MHz, CDCl$_3$, 25°C, TMS): δ (ppm) = 10.20 (s, 1H), 7.88 (m, 4H), 7.35 (m, 4H), 7.10 (m, 1H), 2.38 (s, 3H); IR (KBr): ν (cm^{-1}) = 3351, 3057, 2916, 1649, 1596, 1524, 1438, 1320, 1260, 885, 747, 588; MS (EI, 70eV): m/z: 211[M]$^+$.

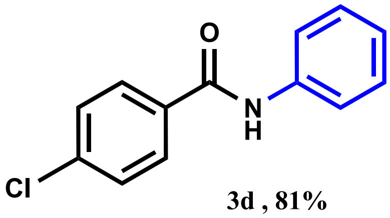

3d , 81%

4-chloro-N-phenylbenzamide (3d)[2]: white solid 81% isolated yield; Mp: 197-200°C; ^{1}H-NMR (500 MHz, CDCl$_3$, 25°C, TMS): δ (ppm) =10.33 (s, 1H), 8.01 (m, 2H), 7.79 (m, 2H), 7.60 (m, 2H), 7.36 (m, 2H), 7.11 (m, 1H); IR (KBr): ν (cm^{-1}) = 3351, 3083, 3056, 1653, 1597, 1528, 1438, 1324, 1258, 1092, 847, 755, 589.

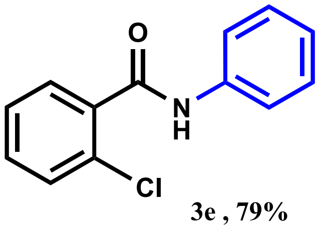

3e , 79%

2-chloro-N-phenylbenzamide (3e)[3]: white solid 79% isolated yield; Mp: 150-151°C; ^{1}H-NMR (500 MHz, $CDCl_3$, 25°C, TMS): δ (ppm) = 10.52 (s, 1H), 7.76 (d, J = 5 Hz, 2H), 7.59 (m, 2H), 7.50 (m, 2H), 7.37 (t, J = 5 Hz, 2H), 7.13 (t, J = 5 Hz, 1H); IR (KBr): ν (cm^{-1}) = 3238, 3187, 3134, 3078, 2980, 1651, 1599, 1546, 1488, 1329, 1262, 1123, 1049, 768, 691.

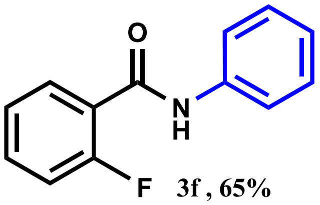

3f , 65%

2-fluoro-N-phenylbenzamide (3f): White solid; 65% isolated yield; Mp:112-114°C; ^{1}H NMR (500 MHz, DMSO-d_6): δ (ppm) = 10.4 (s, 1H), 7.73 (d, J=7.9 Hz, 2H), 7.67 (t, J=7.2Hz, 1H), 7.58 (dd, J=11, 6.2 Hz, 1H), 7.33-7.39 (m, 4H), 7.11 (t, J=7.3Hz, 1H); IR (KBr): (cm^{-1}) = 3316, 1655, 1534, 1451, 1325, 1260, 1194, 1098; MS (EI, 70eV): m/z: 216.08$[M]^+$.

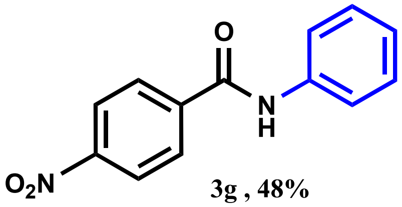

3g , 48%

4-nitro-N-phenylbenzamide (3g)[4]: white solid; 48% isolated yield; Mp: 181-182°C; ^{1}H-NMR (500 MHz, $CDCl_3$, 25°C, TMS): δ (ppm) = 10.93 (s, 1H), 8.33 (d, J = 10 Hz, 2H), 8.27 (d, J = 10 Hz, 2H), 7.85 (d, J = 10 Hz, 2H), 7.36 (t, J = 5 Hz, 2H), 7.13 (t, J = 5 Hz, 1H); IR (KBr): ν (cm^{-1}) = 3321, 3110, 1621, 1592, 1523, 1407, 1351, 1317, 1262, 1105, 832, 724, 506.

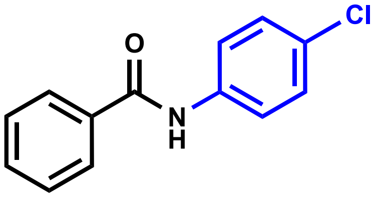

3h , 73%

N-(4-chlorophenyl)benzamide (3h): White solid; 73% isolated yield; Mp: 193-195 °C; ^{1}H NMR (500 MHz, DMSO-d6): δ (ppm) = 10.37 (s, 1H), 7.93 (d, J=7.5Hz, 2H), 7.8 (d, J=8.6Hz, 2H), 7.58 (t, J=7.0Hz, 1H), 7.52 (t, J=7.4Hz, 2H), 7.39 (d, J=8.6Hz, 2H); IR (KBr): ν (cm^{-1}) = 3346, 1651, 1595, 1397, 1275, 750; MS (EI, 70eV): m/z: 231$[M]^+$.

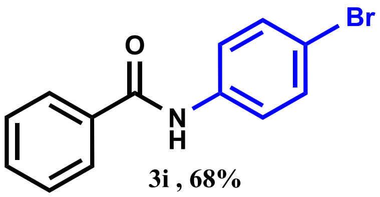

3i , 68%

N-(4-Chlorophenyl)benzamide (3i): white solid; 68% isolated yield, Mp: 193-194 °C; ^{1}H NMR (500 MHz, $CDCl_3$): δ (ppm) = 7.86-7.85 (d, 3J=8.0 Hz, 2H), 7.79 (s, 1H), 7.61-7.58 (m, 2H), 7.56-7.55 (m, 1H), 7.51-7.48 (m, 2H), 7.34-7.33 (d, 3J=8.0 Hz, 2H); IR (KBr): ν (cm^{-1}) = 3348, 1655, 1596, 1519, 1396, 1312, 1250, 1092, 824, 716, 647; MS (EI, 70eV): m/z: 275$[M]^+$.

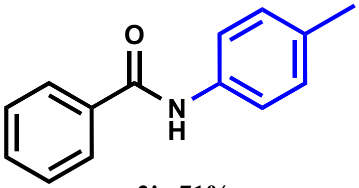

3j , 71%

N-(p-Tolyl)benzamide (3j): white solid; 71% isolated yield, Mp: 159-160 °C; ^{1}H NMR (500 MHz, $CDCl_3$): δ (ppm) = 7.87-7.86 (d, 3J=7.9 Hz, 2H), 7.74 (s, 1H), 7.56-7.54 (m, 5H), 7.18-7.17 (d, 3J=7.9 Hz, 1H), 2.34 (s, 3H); IR (KBr): ν (cm^{-1})= 3309, 1646, 1596, 1578, 1510, 1317, 1265, 813, 694; MS (EI, 70eV): m/z: 211$[M]^+$.

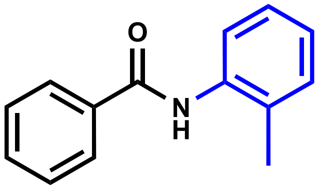

3k , 86%

N-(o-tolyl)benzamide (3k): White solid; 86% isolated yield; Mp: 143-145 °C; ^{1}H NMR (500 MHz, DMSO-d_6): δ (ppm) = 9.89 (s, 1H), 8.00 (d, J=7.8Hz, 2H), 7.62-7.58 (m, 1H), 7.53 (dd, J=11, 3.9 Hz, 2H), 7.36 (d, J=7.7 Hz, 1H), 7.28 (d, J=7.3 Hz, 1H), 7.23 (t, J=7.5Hz, 1H), 7.18 (t, J=7.4Hz, 1H), 2.5 (s, 3H);^{13}C NMR (101 MHz, $CDCl_3$): δ (ppm) =165.6 (CO amide), 135.8 (C), 135.1 (C), 131.7 (CH), 130.5 (CH), 129.3 (C), 128.6 (CH), 127.1 (CH), 126.7 (CH), 125.4 (CH), 123.2 (CH), 17.8 (CH_3); IR (KBr): ν (cm^{-1})= 3244, 3057, 2923, 1648, 1524, 1487, 1308, 1274, 747; MS (EI, 70eV): m/z: 211.1$[M]^+$.

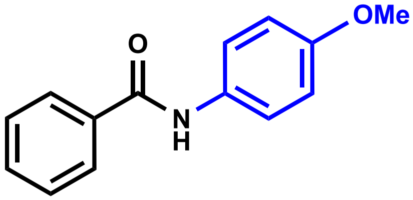

3l ,78%

N-(4-methoxyphenyl)benzamide (3l): Brown solid; 78% isolated yield; Mp: 152-155 °C; ^{1}H NMR (500 MHz, DMSO-d_6): δ (ppm) = 10.13 (s, 1H), 7.95 (d, J=4.9 Hz, 2H), 7.68 (d, J=8.2Hz, 2H), 7.58-7.54 (m, 1H), 7.51 (t, J=7.3Hz, 2H), 6.92 (d, J=7.8Hz, 2H), 3.76 (s, 3H); ^{13}C NMR (75 MHz, DMSO): δ (ppm) = 166.1 (C),

155.7 (C), 135.1 (C), 132.3 (C), 131.3 (CH), 128.3 (CH), 127.5 (CH), 122.1 (CH), 113.8 (CH), 55.7 (CH_3); IR (KBr): ν(cm^{-1})= 3327, 2960,1643, 1512,1405, 1268, 1029, 823; MS (EI, 70eV): m/z: 227.1$[M]^+$.

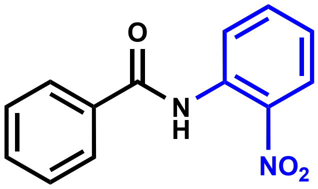

3m , 54%

N-(2-Nitrophenyl)benzamide (3m)[5]: Yellow solid; 54% Isolated yield; Mp:75°C; ^{1}H NMR (500 MHz, $CDCl_3$): δ (ppm) = 11.36 (s, 1 H, NH), 9.03 (d, J = 8.6 Hz, 1 H), 8.30 (d, J = 10.0 Hz, 1 H), 8.01 (d, J = 7.1 Hz, 2 H), 7.73 (t, J = 8.6 Hz, 1 H), 7.62 (t, J = 8.6 Hz, 1 H), 7.56 (dd, J = 8.6, 7.1 Hz, 2 H), 7.24 (dd, J = 10.0, 8.6 Hz, 1 H); ^{13}C NMR (101 MHz, CDCl3): δ (ppm) = 165.8, 136.5, 136.2, 135.4, 134.0, 132.7, 129.1, 127.4, 125.9, 123.3, 122.1; IR (KBr): ν (cm^{-1}) =3349, 1690, 1489, 1332, 739, 695; MS (EI, 70eV): m/z: 243$[M]^+$.

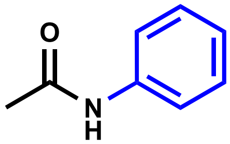

3n , 85%

N-phenylacetamide (3n)[6]: white solid; 85% Isolated yield; Mp: 115 °C; ^{1}H NMR (400 MHz, DMSO-d_6): δ (ppm) = 10.27 (s, 1H), 7.97 (d, J = 6.9 Hz, 2H), 7.80 (d, J = 7.6 Hz, 2H), 7.59 (t, J = 7.2 Hz, 1H), 7.53 (t, J = 7.2 Hz, 2H), 7.38 – 7.33 (m, 2H), 7.10 (t, J = 7.4 Hz, 1H); ^{13}C NMR (101 MHz, $CDCl_3$): δ (ppm) = 168.8, 138.1, 129.1, 124.4, 120.1, 24.6; IR (KBr): ν (cm^{-1}) = 3293, 2924, 2855, 1662, 1603, 1549, 1495, 1433, 1317, 1259, 753.

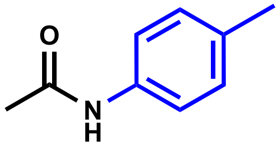

3o ,78%

N-p-tolylacetamide (3o): white solid; 78% Isolated yield; Mp: 151-152 °C; ^{1}H NMR: (500 MHz, DMSO-d_6, 25 °C, TMS): δ (ppm) = 7.37 (d, J = 8.25 Hz, 2H), 7.26 (s, 1H), 7.1 (d, J = 8.05 Hz, 2H), 2.3 (s, 3H), 2.15 (s, 3H). ^{13}C NMR (125 MHz, DMSO-d_6, 25 °C, TMS): δ (ppm) = 168.32, 135.33, 133.93, 129.52, 120.06, 24.46, 20.83; IR (KBr) ν (cm^{-1}) = 3296, 3193, 2930, 2863, 1663, 1544, 1451, 1317, 1261, 820.

4. ^{1}H NMR and ^{13}CNMR spectra of product:

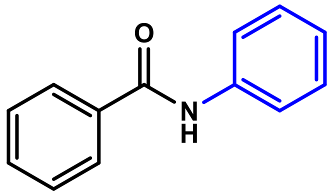

3a ,91% X= I
3b 48% X=Cl

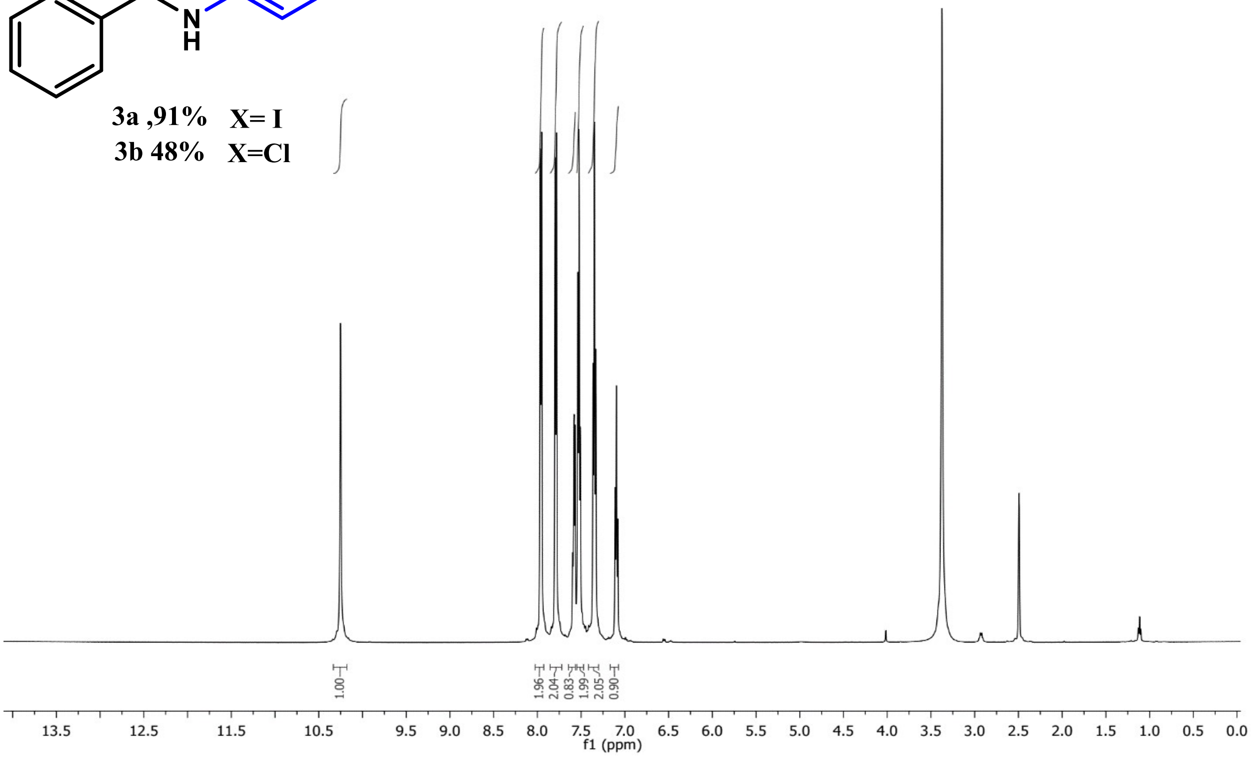

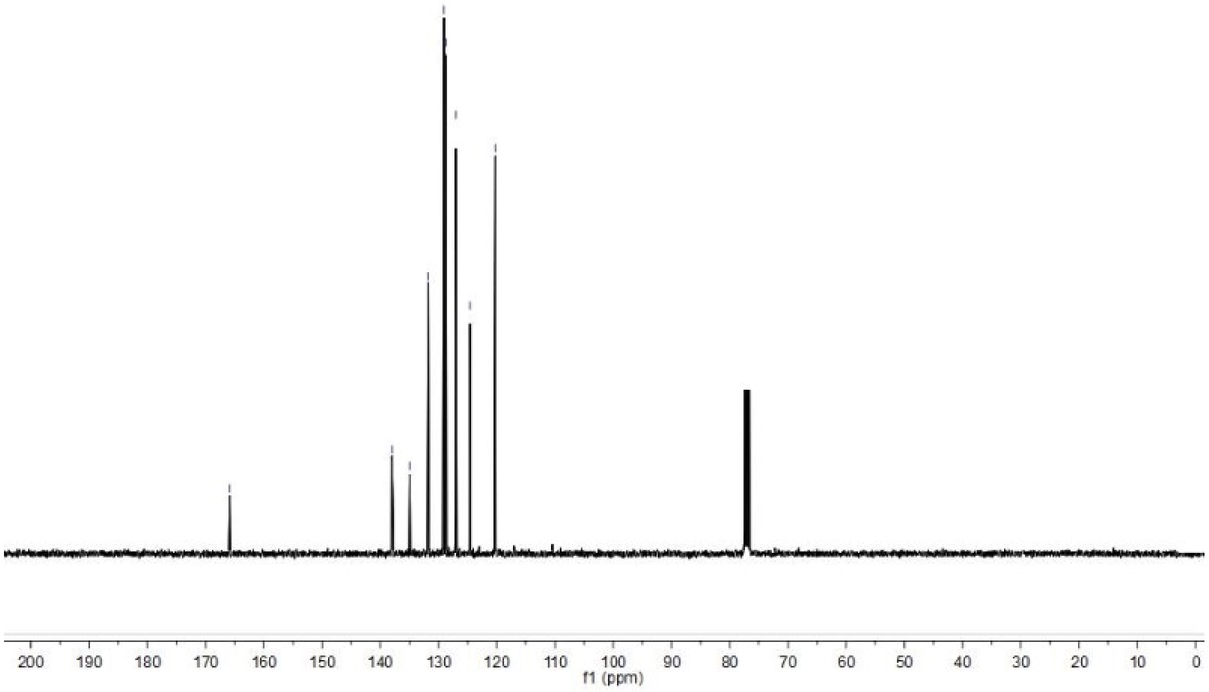

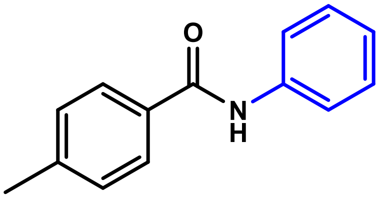

3c,82%

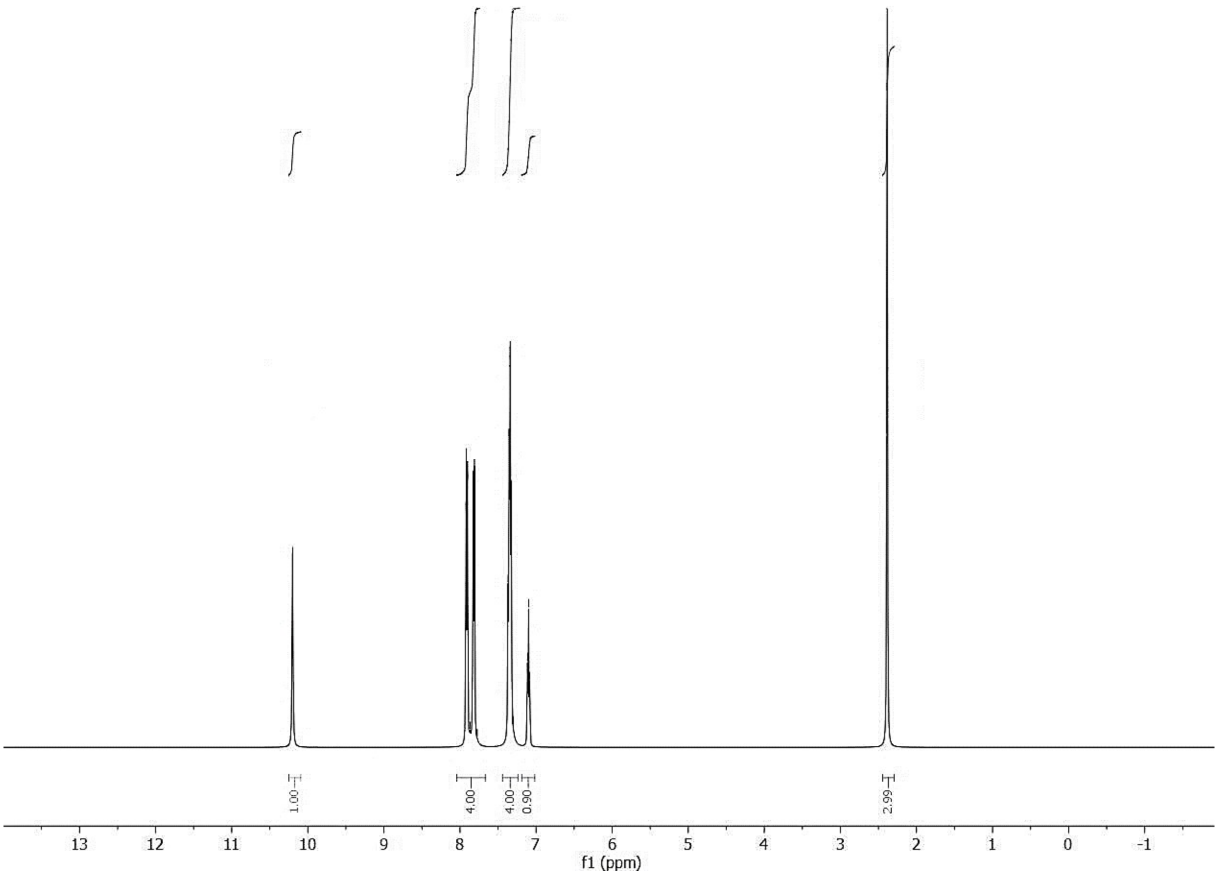

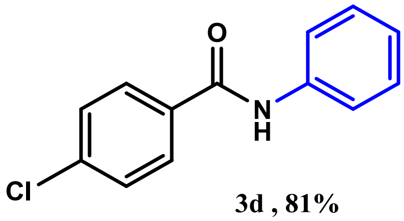

3d , 81%

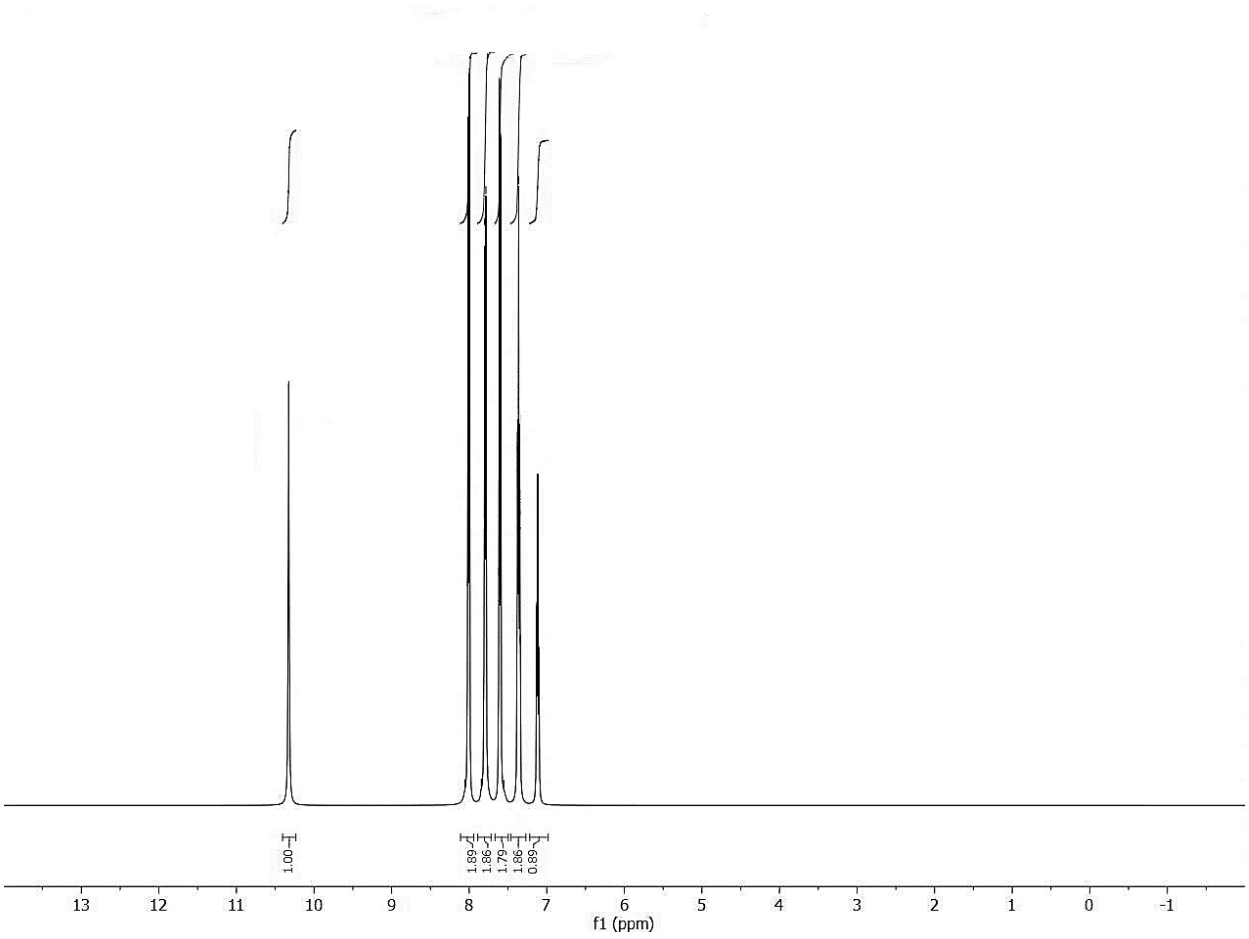

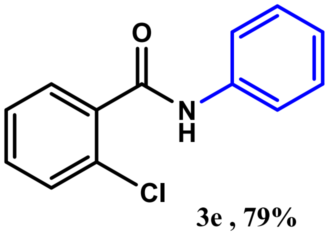
O
N
H
Cl

3e , 79%

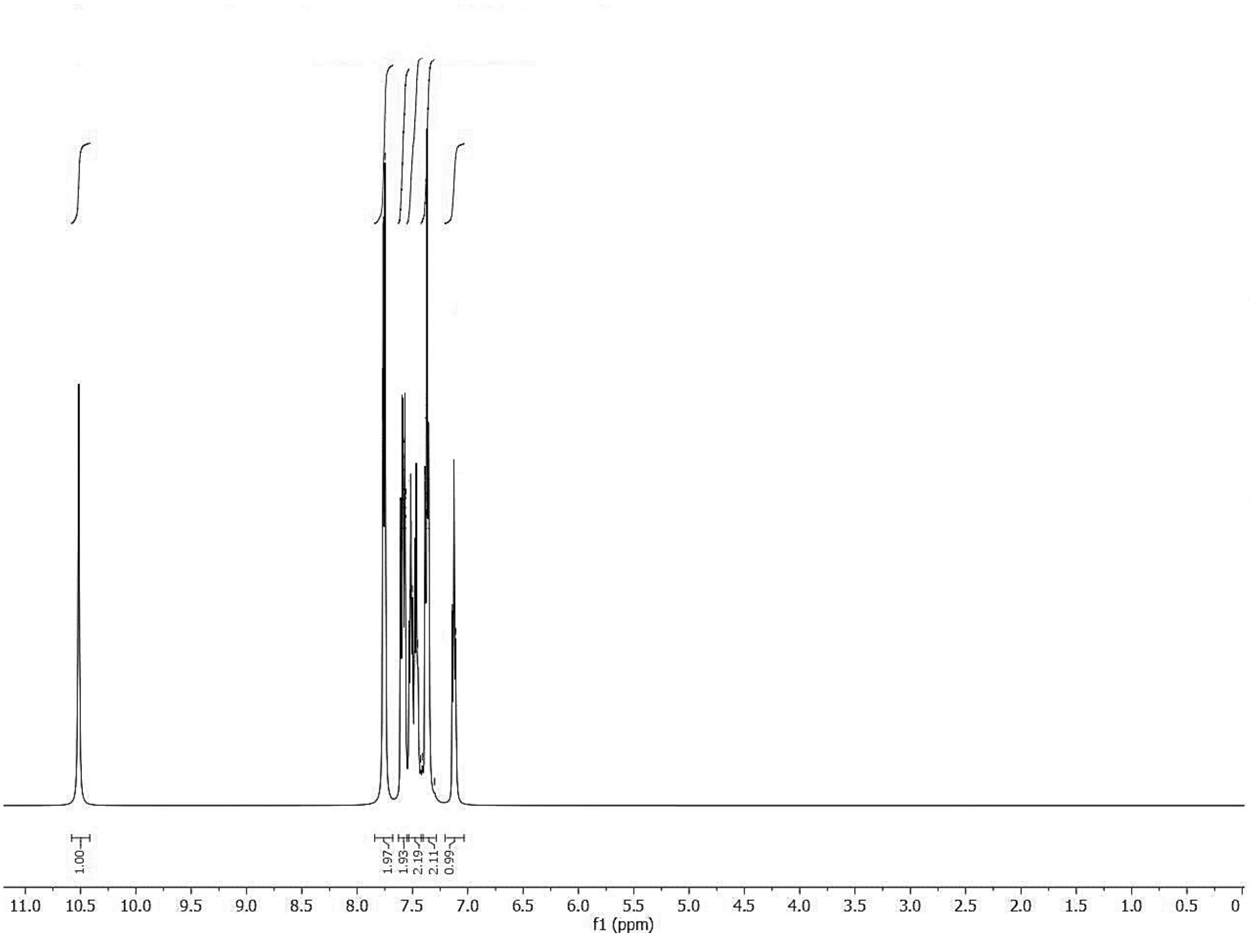
1.00
1.97
1.93
2.19
2.11
0.99
11.0
10.5
10.0
9.5
9.0
8.5
8.0
7.5
7.0
6.5
6.0
5.5
5.0
4.5
4.0
3.5
3.0
2.5
2.0
1.5
1.0
0.5
0
f1 (ppm)

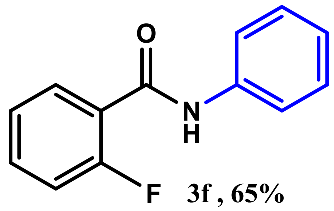

3f , 65%

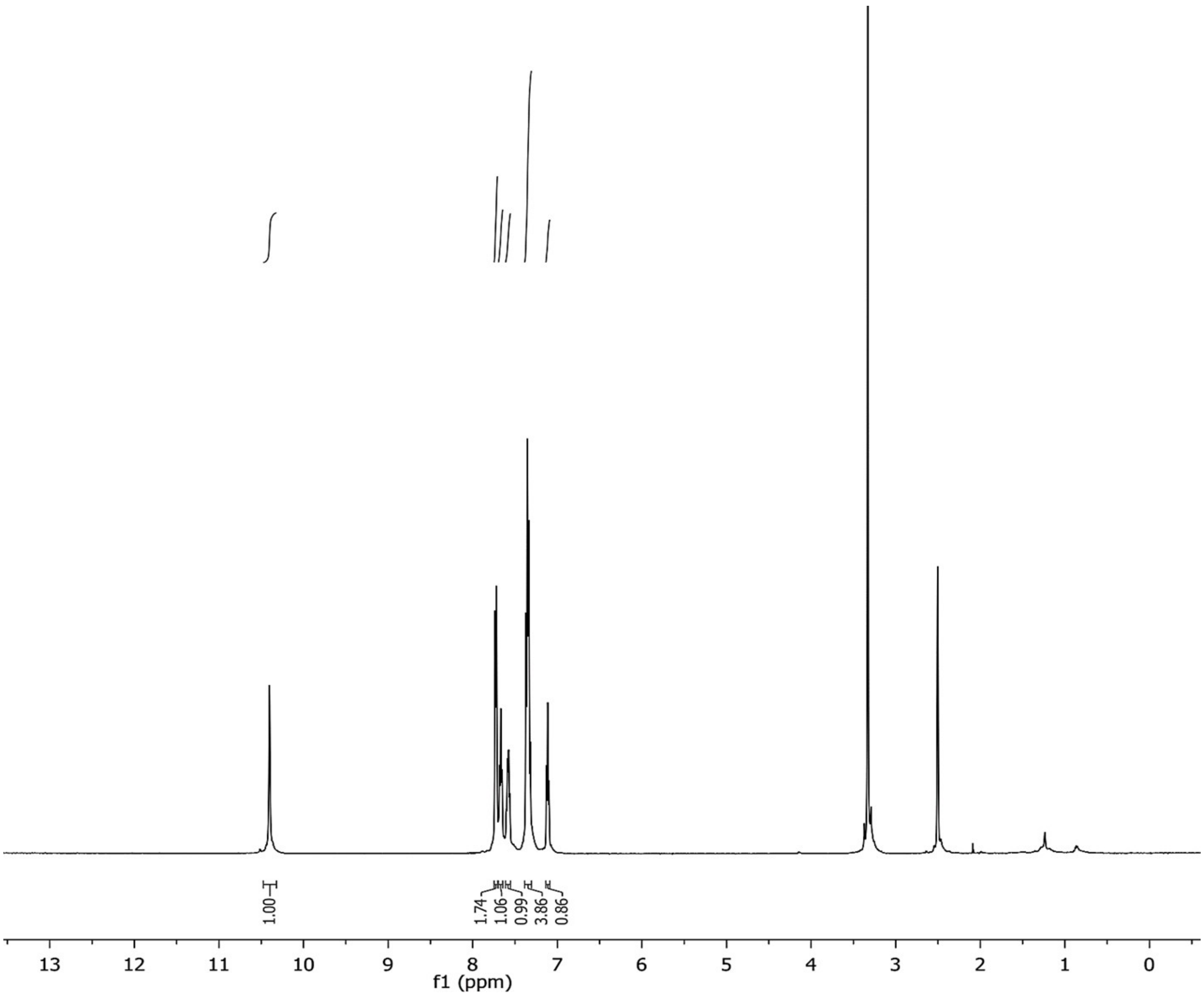

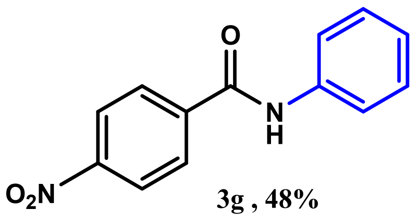

3g , 48%

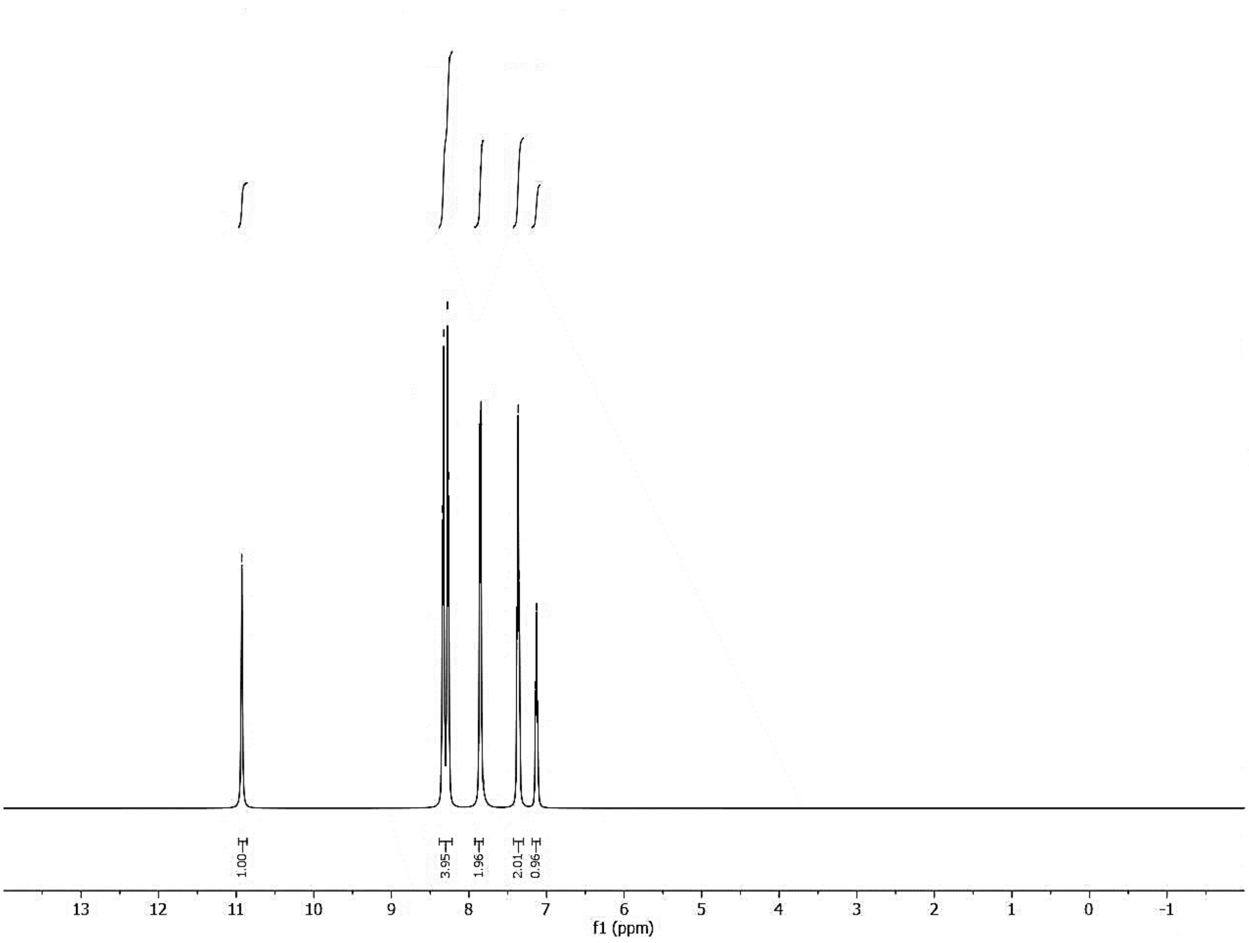

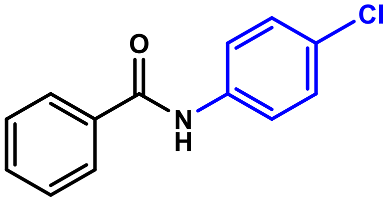
O
N
H
Cl

3h , 73%

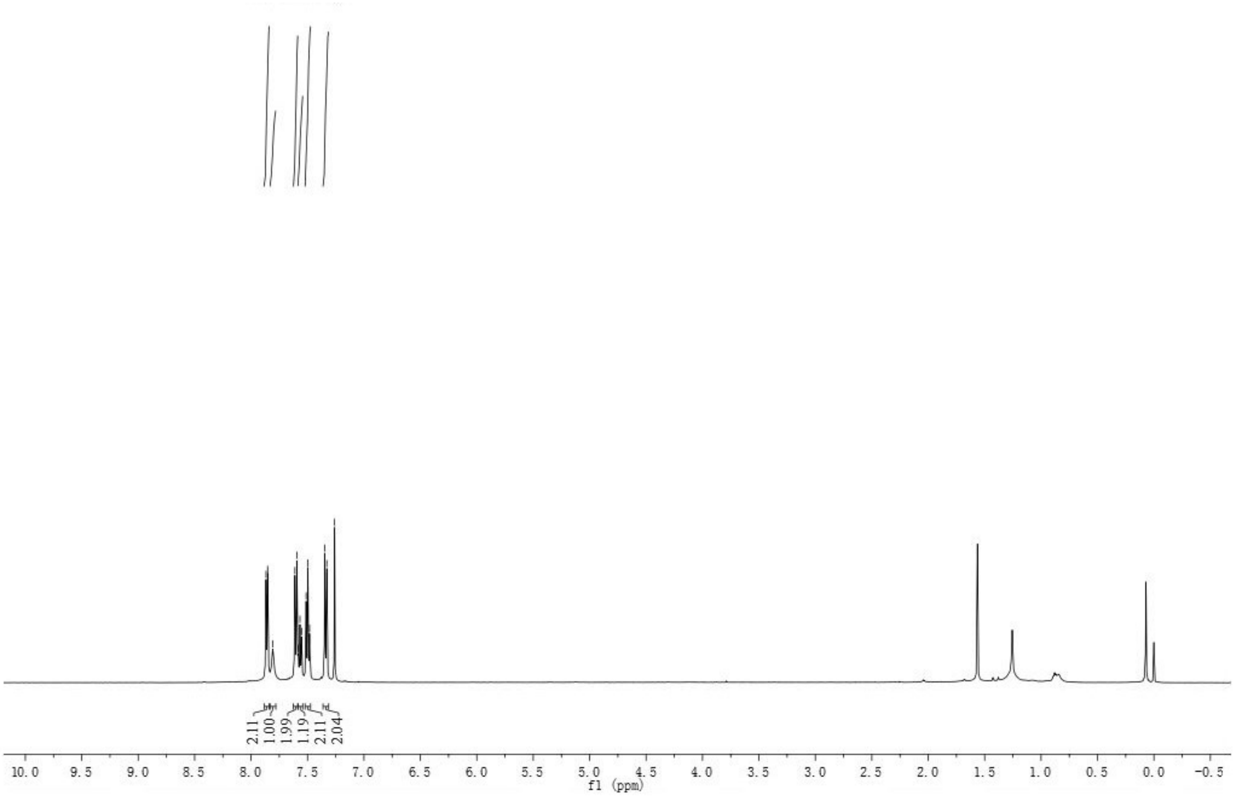
2.11
1.00
1.99
1.19
2.11
2.04
10.0 9.5 9.0 8.5 8.0 7.5 7.0 6.5 6.0 5.5 5.0 4.5 4.0 3.5 3.0 2.5 2.0 1.5 1.0 0.5 0.0 -0.5
f1 (ppm)

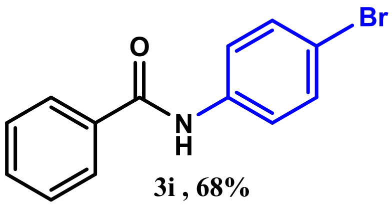

3i , 68%

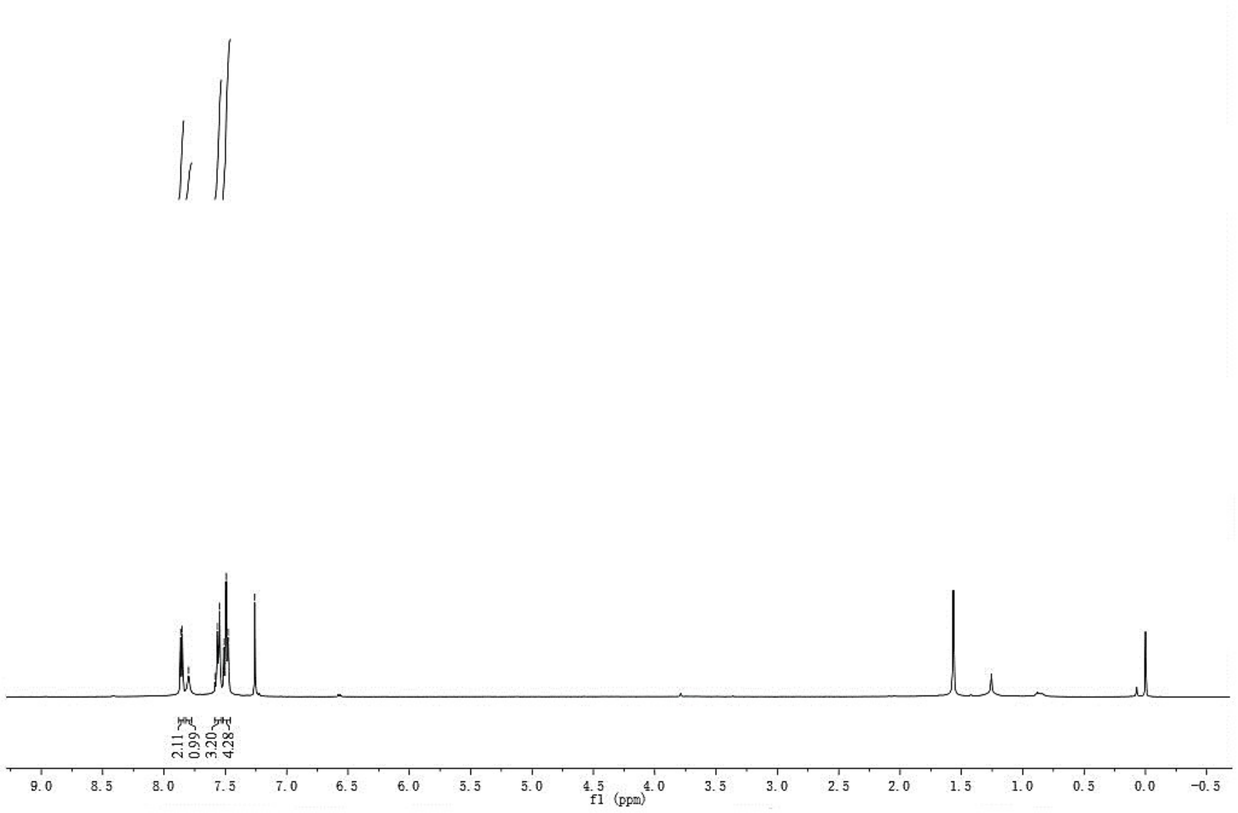

2.11
0.99
3.20
4.28
9.0 8.5 8.0 7.5 7.0 6.5 6.0 5.5 5.0 4.5 4.0 3.5 3.0 2.5 2.0 1.5 1.0 0.5 0.0 -0.5
f1 (ppm)

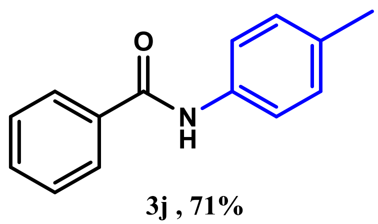

3j , 71%

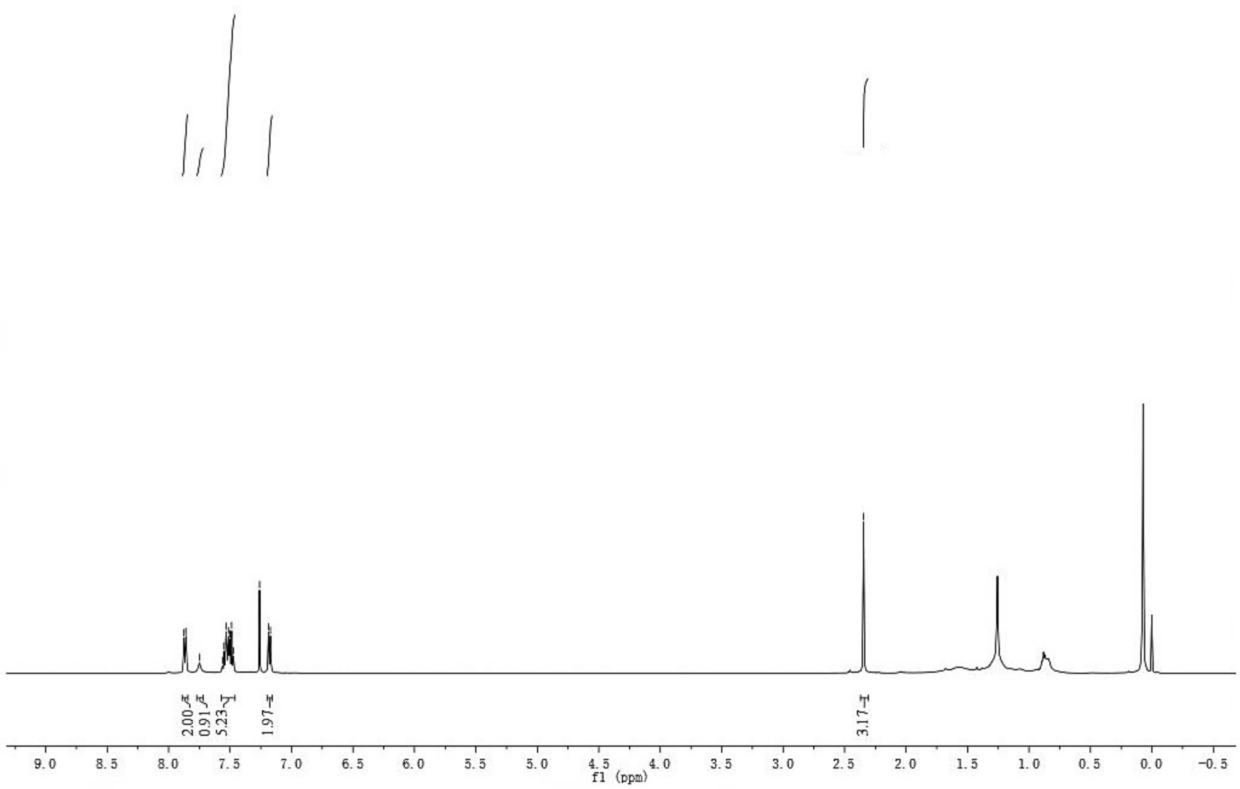
2.00
0.91
5.23
1.97
3.17
9.0
8.5
8.0
7.5
7.0
6.5
6.0
5.5
5.0
4.5
4.0
3.5
3.0
2.5
2.0
1.5
1.0
0.5
0.0
-0.5
f1 (ppm)

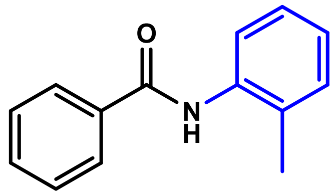

O
N
H

3k , 86%

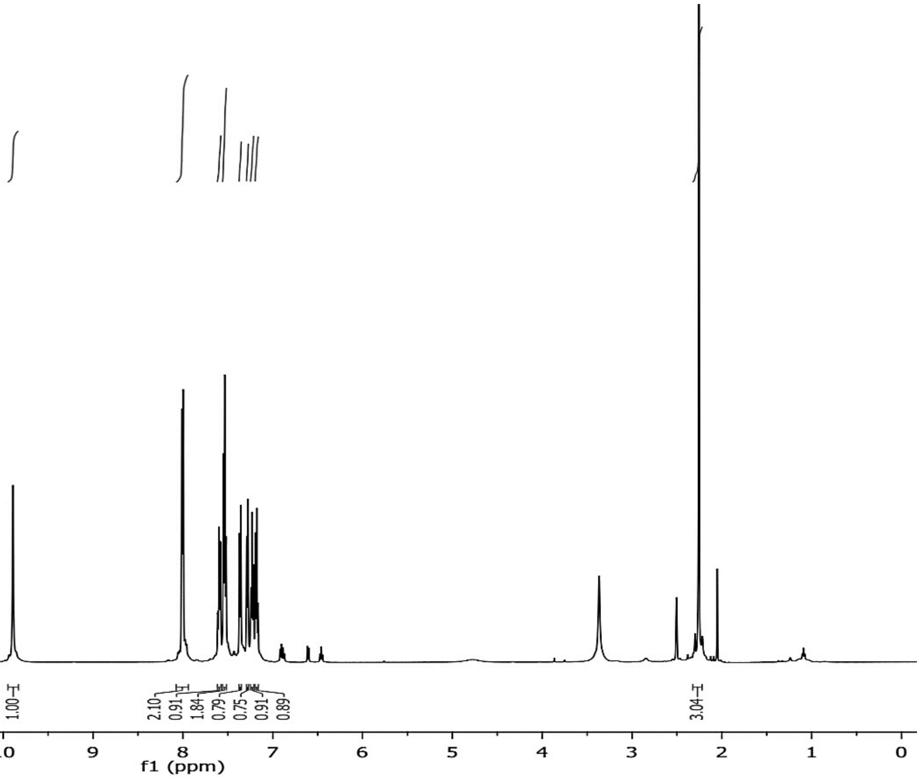

1.00
2.10
0.91
1.84
0.79
0.75
0.91
0.89
3.04
13
12
11
10
9
8
7
6
5
4
3
2
1
0
f1 (ppm)

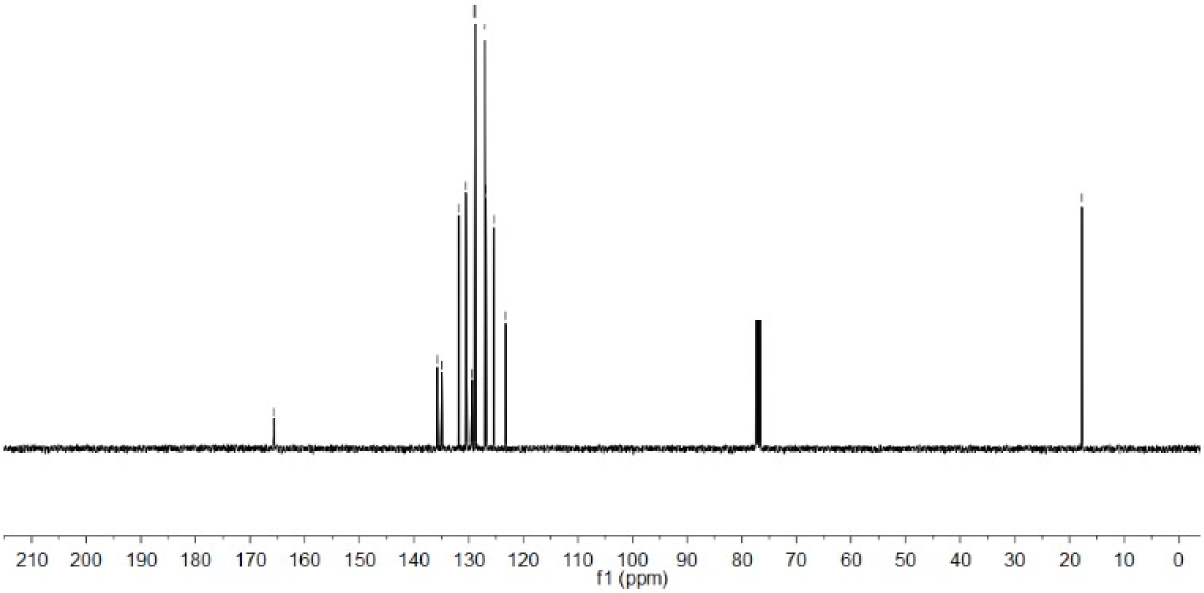

210
200
190
180
170
160
150
140
130
120
110
100
90
80
70
60
50
40
30
20
10
0
f1 (ppm)

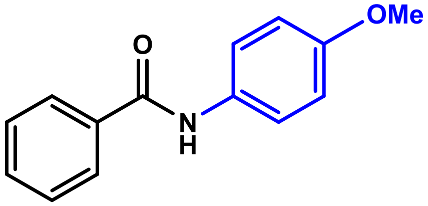
OMe
O
N
H

3l ,78%

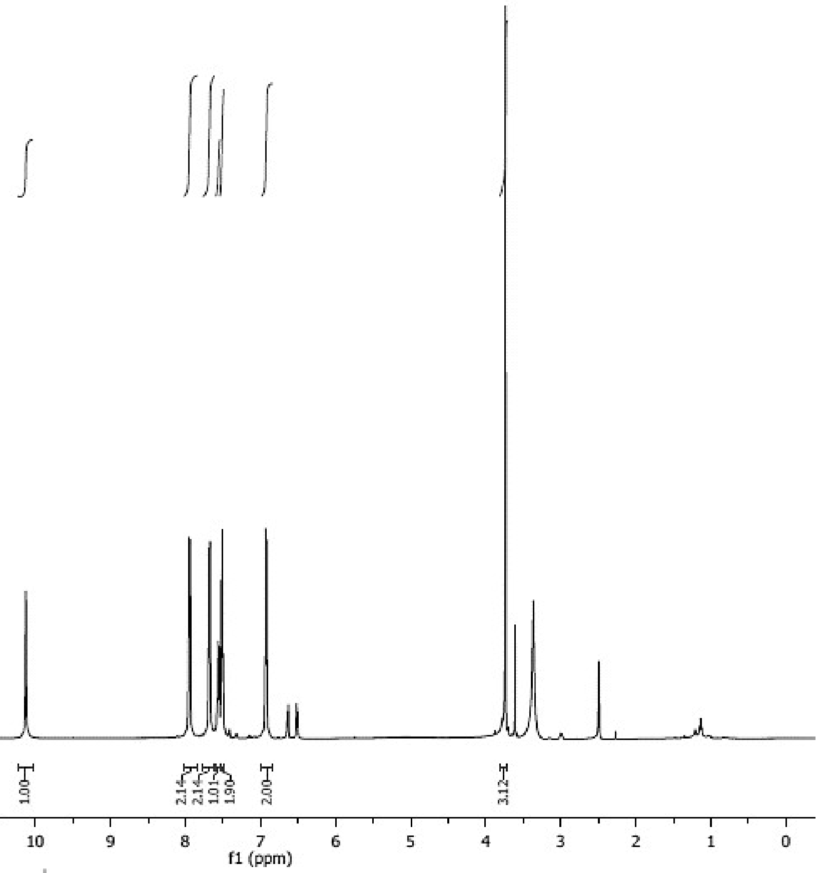
1.00
2.14
2.14
1.01
1.90
2.00
3.12
15 14 13 12 11 10 9 8 7 6 5 4 3 2 1 0
f1 (ppm)

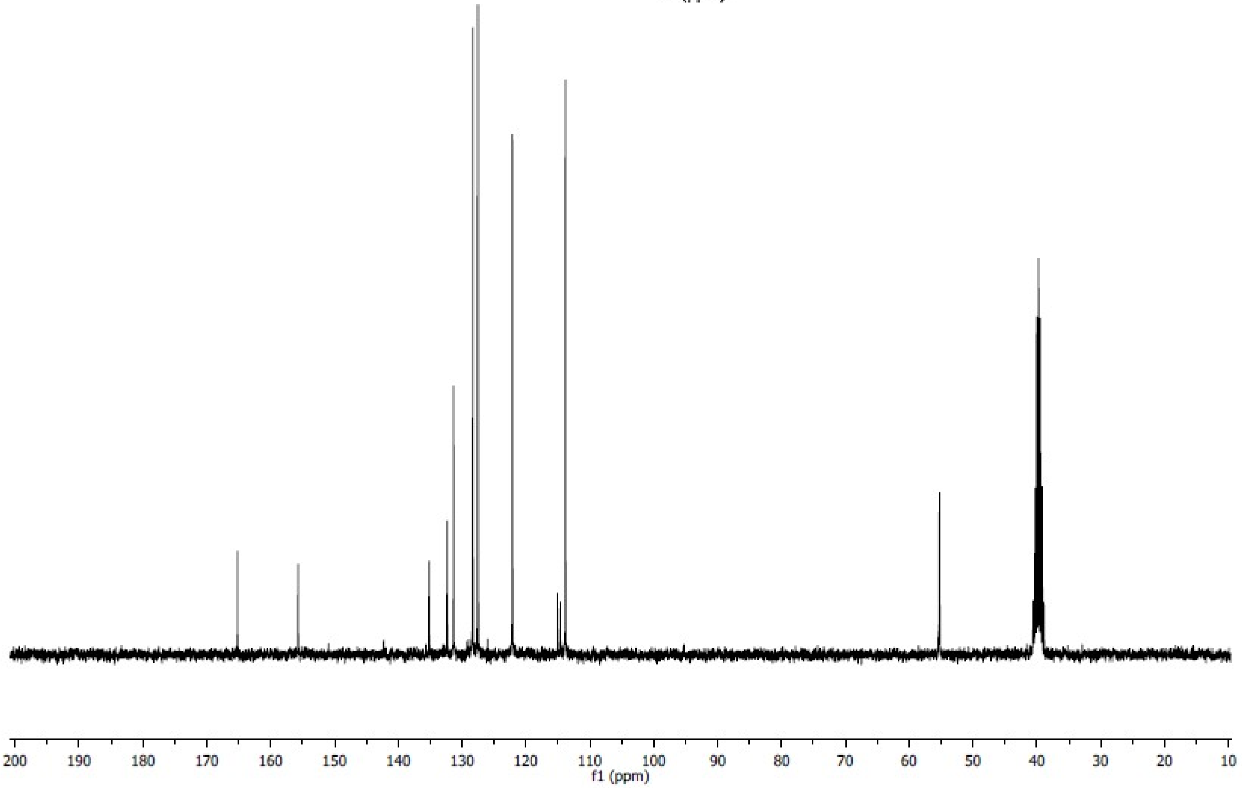
200 190 180 170 160 150 140 130 120 110 100 90 80 70 60 50 40 30 20 10
f1 (ppm)

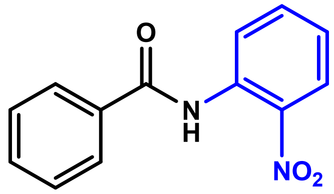
O
N
H
NO2

3m , 54%

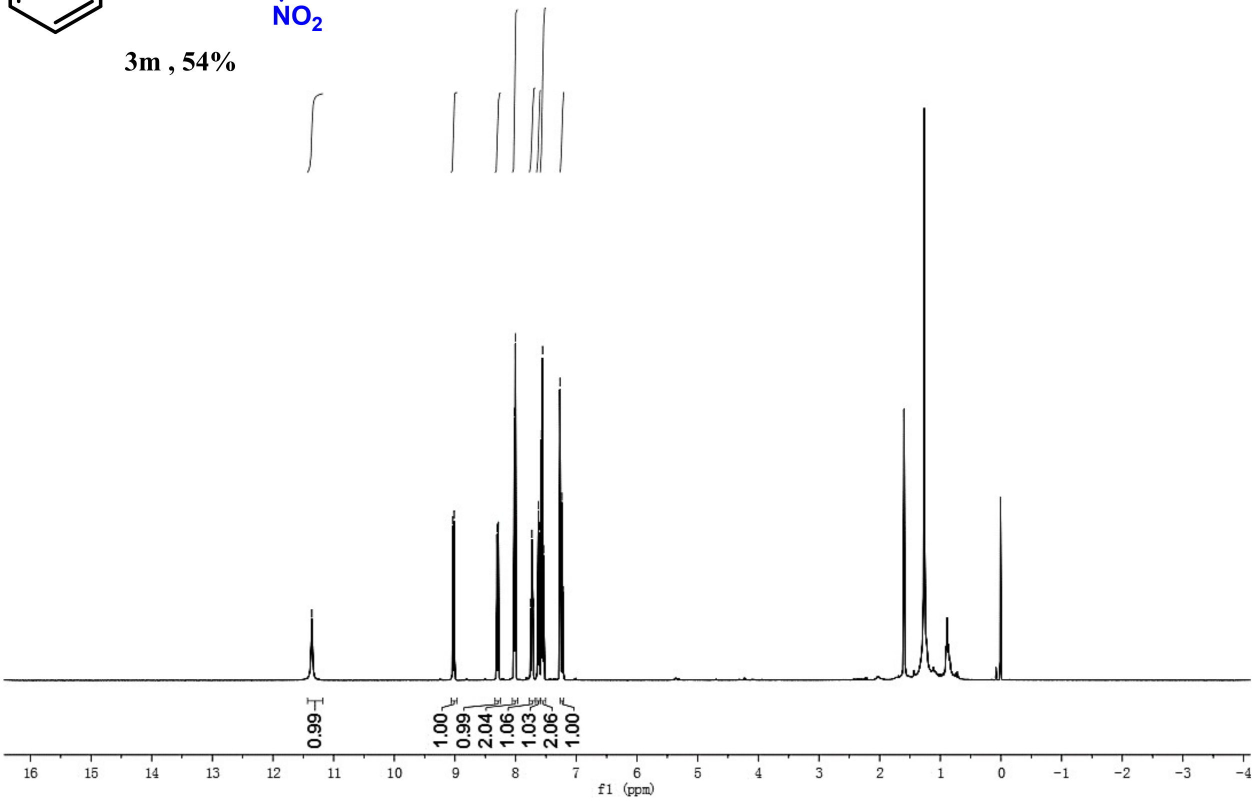
0.99
1.00
0.99
2.04
1.06
1.03
2.06
1.00
16 15 14 13 12 11 10 9 8 7 6 5 4 3 2 1 0 −1 −2 −3 −4
f1 (ppm)

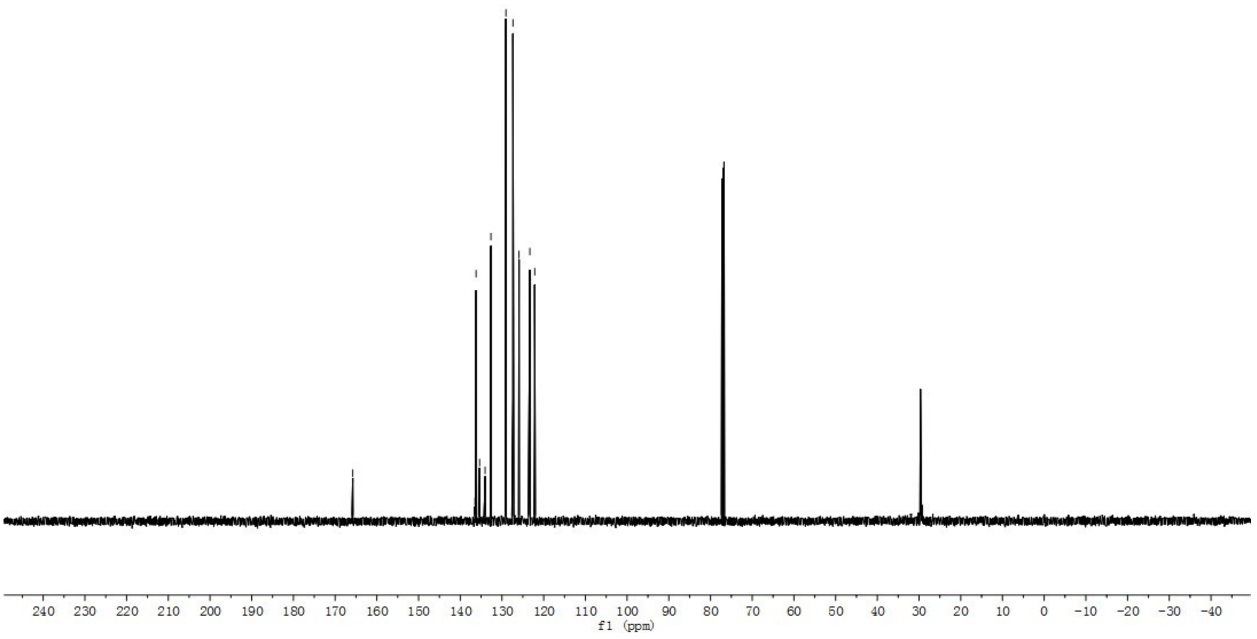
240 230 220 210 200 190 180 170 160 150 140 130 120 110 100 90 80 70 60 50 40 30 20 10 0 −10 −20 −30 −40
f1 (ppm)

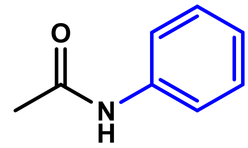
O
N
H

3n , 85%

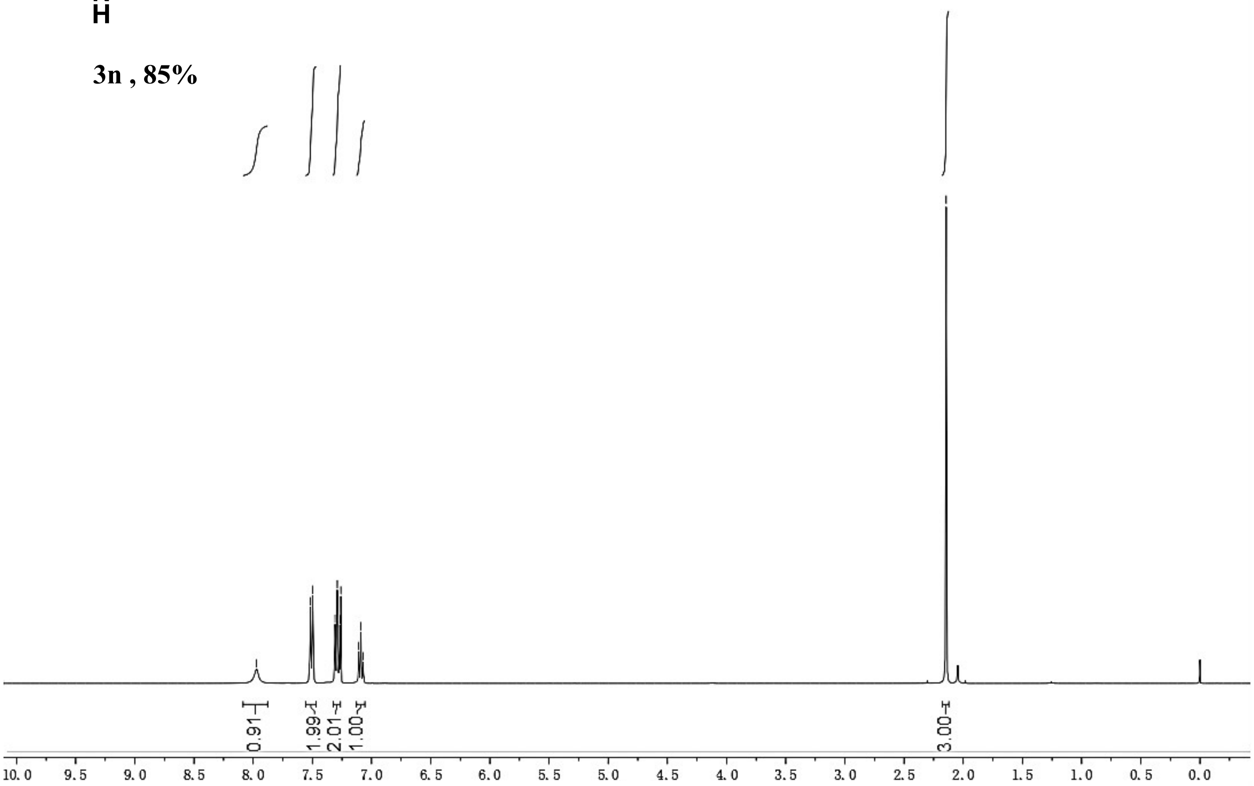
0.91
1.99
2.01
1.00
3.00
10.0 9.5 9.0 8.5 8.0 7.5 7.0 6.5 6.0 5.5 5.0 4.5 4.0 3.5 3.0 2.5 2.0 1.5 1.0 0.5 0.0

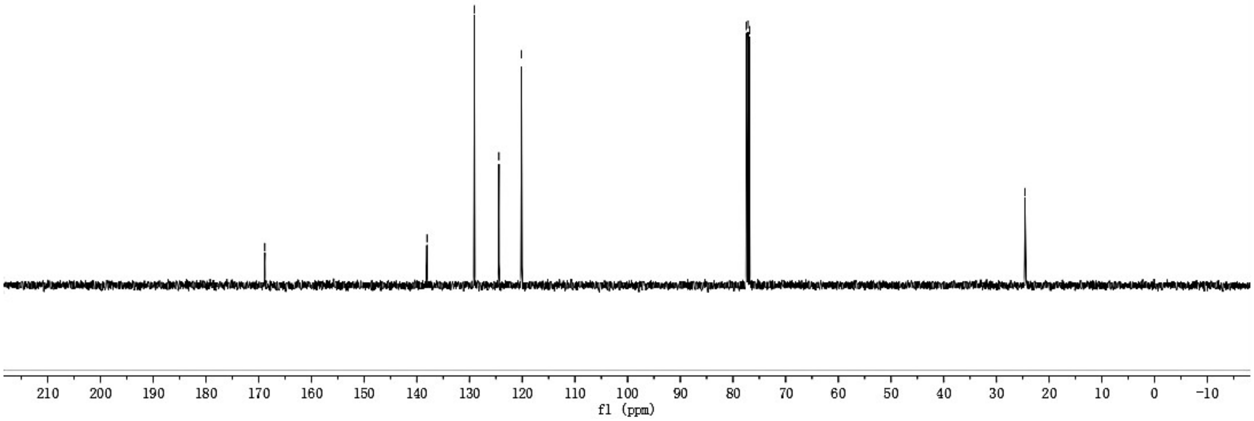
210 200 190 180 170 160 150 140 130 120 110 100 90 80 70 60 50 40 30 20 10 0 -10
f1 (ppm)

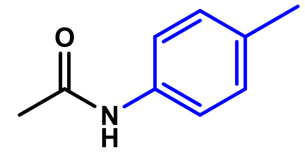
O
N
H

3o ,78%

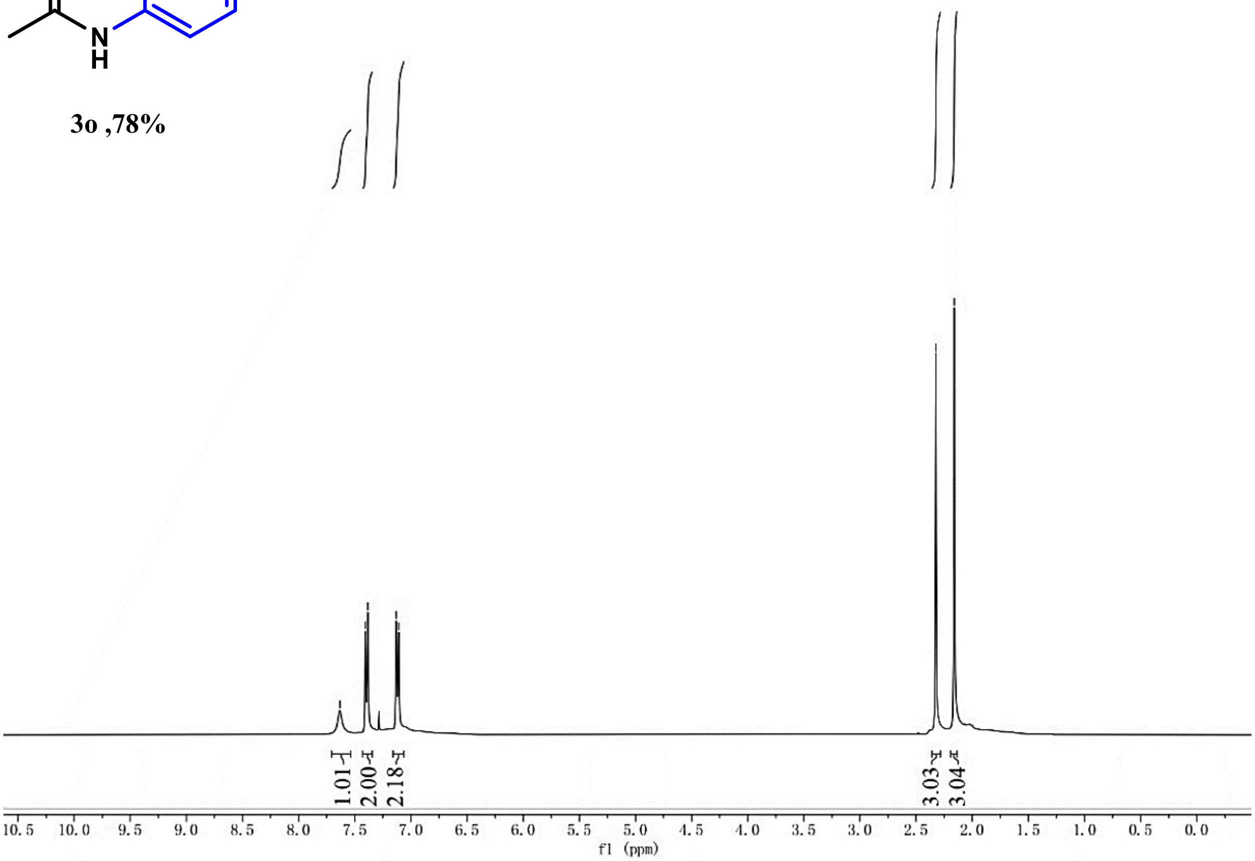
1.01
2.00
2.18
3.03
3.04
10.5 10.0 9.5 9.0 8.5 8.0 7.5 7.0 6.5 6.0 5.5 5.0 4.5 4.0 3.5 3.0 2.5 2.0 1.5 1.0 0.5 0.0
f1 (ppm)

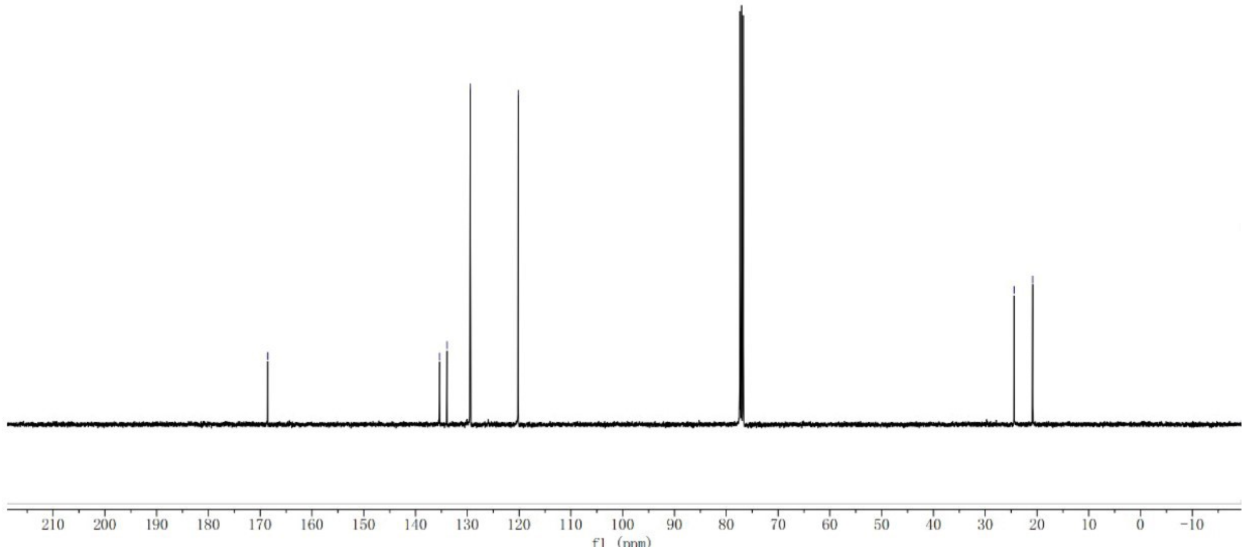
210 200 190 180 170 160 150 140 130 120 110 100 90 80 70 60 50 40 30 20 10 0 −10

References:

(1) Xu, M.; Zhang, X.; Shao, Y.; Han, J.; Zhong, P. The Synthesis of *N* -Arylated Amides *via* Copper(II) Triflate- Catalyzed Direct Oxygenation and *N* -Arylation of Benzylamines with Aryl Iodides. *Adv Synth Catal* **2012**, *354* (14–15), 2665–2670. https://doi.org/10.1002/adsc.201200424.

(2) Moon, H.; Lee, S. Reductive Cross-Coupling of N-Acyl Pyrazole and Nitroarene Using Tetrahydroxydiboron: Synthesis of Secondary Amides. *Organic & Biomolecular Chemistry* **2023**, *21* (41), 8329–8334.

(3) Du, J.; Luo, K.; Zhang, X. Synthesis of Amides through an Oxidative Amidation of Tetrazoles with Aldehydes under Transition-Metal-Free Conditions. *RSC advances* **2014**, *4* (97), 54539–54546.

(4) Wang, Y.-J.; Zhang, G.-Y.; Shoberu, A.; Zou, J.-P. Iron-Catalyzed Oxidative Amidation of Acylhydrazines with Amines. *Tetrahedron Letters* **2021**, *80*, 153316.

(5) Depa, M. R.; Potla, S.; Narkhede, U. C.; Jadhav, V. D.; Vidavalur, S. Copper-Mediated Regioselective Efficient Direct Ortho-Nitration of Anilide Derivatives. *Tetrahedron Letters* **2020**, *61* (33), 152223.

(6) Liu, D.; Fan, Y.; Liu, M.; Ge, Q.; Gao, R.; Cong, H. Cucurbit[7]Uril-Catalyzed Beckmann Rearrangement of Arylketoximes. *Org. Lett.* **2024**, *26* (18), 3896–3900. https://doi.org/10.1021/acs.orglett.4c01061.
